# Efficacy and safety of traditional Chinese medicine for cancer-related fatigue: a systematic literature review of randomized controlled trials

**DOI:** 10.1186/s13020-023-00849-y

**Published:** 2023-11-01

**Authors:** Jingya Yang, Yuxiao Li, Chi Ian Chau, Junnan Shi, Xianwen Chen, Hao Hu, Carolina Oi Lam Ung

**Affiliations:** 1 State Key Laboratory of Quality Research in Chinese Medicine, Institute of Chinese Medical Sciences, University of Macau, Macao, SAR China; 2 Department of Public Health and Medicinal Administration, Faculty of Health Sciences, University of Macau, Macao, SAR China

**Keywords:** Cancer-related fatigue, Traditional Chinese medicine, RCTs, CONSORT-CHM, Risk of bias

## Abstract

**Background:**

Cancer-related fatigue (CRF) is an extremely common and long-term condition that affects the physical and mental health of oncology patients. While the treatment for CRF with western medicine and non-pharmacological therapy remains uncertain and challenging, traditional Chinese medicine (TCM) has become a trending option for the patients. Based on the findings from randomized controlled trials (RCTs), this study aims to identify and evaluate the evidence about the efficacy and safety of TCM for CRF.

**Methods:**

A systematic literature search was conducted according to the PRISMA literature research guidelines. Seven electronic databases including PubMed, the Cochrane Library, Embase, Web of Science, Scopus, China National Knowledge Infrastructure (CNKI) and Wanfang database were searched to identify RCTs which investigated TCM in the treatment of CRF published since inception to December 2022. RCTs comparing TCM with no treatment, placebo, or pharmacological interventions were considered eligible for this review. The Consolidated Standards of Reporting Trials Statement extensions for Chinese herbal medicine Formulas (CONSORT-CHM) and the Cochrane Collaboration’s Risk of Bias tool were used in this review to evaluate the quality and the risk of bias of all included trials.

**Results:**

A total of 82 RCTs were included in this review, regardless of whether they were published in English or Chinese. After data extraction and results evaluation, 78 trials demonstrated overall efficacy in using TCM for CRF patients compared with the control group, in which 33 trials showed that the efficacy rate was statistically significant (p < 0.05 or p < 0.01). TCM was also shown to be beneficial in improving the scores of relevant scales (e.g., PFS, QoL, TCM syndrome score, other fatigue scales etc.) or physical tests indicators (e.g., cytokines, blood test etc.). The most common herbs found in Chinese medicine were *Astragali Radix*, *Ginseng Radix* and *Codonopsis Radix*. Some TCM products, such as Kangai Injection, Buzhong Yiqi Decoction and Shenqi Fuzheng Injection could provide a reference for medication in this review. A range of non-serious, reversible adverse effects associated with the use of TCM was also reported. However, the result of evaluation showed that none of the trials fully met all the CONSORT-CHM criteria, the quality of included trials was generally poor and the risk of bias was mostly uncertain.

**Conclusion:**

There is some evidence supporting the efficacy and safety of TCM in managing CRF in this systematic review. However, no clear conclusion can be made due to the inadequate reporting of efficacy and adverse reactions. In view of some concerns about the existing evidence after the evaluation, it is essential to standardize the comprehensive identification and efficacy measurement standards, improve the quality of RCTs and conduct more multicomponent therapies to provide an updated reference for CRF patients medication in the future.

The protocol of this systematic review has been registered on PROSPERO (CRD42023413625). [https://www.crd.york.ac.uk/prospero/display_record.php?ID=CRD42023413625].

**Supplementary Information:**

The online version contains supplementary material available at 10.1186/s13020-023-00849-y.

## Background

Cancer-related fatigue (CRF) refers to a persistent, subjective sense of tiredness or exhaustion related to cancer or cancer treatment that is not proportional to recent activity and interferes with usual functioning [1]. Underlying cancer pathology, treatment toxicity, physiological abnormalities resulting from complications (including anemia, infection, nausea etc.), or psychological factors can all be predisposing factors for CRF [2]. At present, CRF is extremely common imposing long-term impacts on the physical and mental health of patients, with some reports estimating a prevalence up to 60% [3, 4].

Currently, there is not a standard approach to CRF treatment. Western medicine therapies and non-pharmacological therapies have been commonly employed to help alleviate the fatigue experienced by oncology patients [2, 5]. The recommended western medicines include hematopoietic drugs, antidepressants, psychostimulants, analgesic, etc. [2]. Some patients reportedly benefit from exercise, acupuncture, massage, nutrition, sleep, education or other cognitive–behavioral interventions [5–8]. Nevertheless, the clinical use of these therapies has been considered controversial due to a lack of adequate systematic assessment and strong evidence.

Apart from western medicines and non-pharmacological therapies, traditional Chinese medicines (TCM) have attracted extensive attention for patients in managing CRF during their fight against cancer [9]. TCM has a long history and is famous worldwide for its theories. According to the clinical symptoms, CRF is recognized as a deficiency pattern [10], including both *qi* and blood deficiency, disharmony of *yin* and *yang*, hypofunction of liver, kidney, spleen or other organs [11]. Common herbs used in TCM such as *Astragalus, Turmeric, Ginseng* have been shown to be beneficial in relieving CRF and pain as well as improving immune system function [9]. Decoctions of TCM used for CRF often contain complex and multiple pharmacological activities at various targets. For instance, *Buzhong Yiqi Decoction* contains at least eight herbs, and its therapeutic effects could be attributed to the processes such as interfering with tumor cell proliferation, inducing tumor cell apoptosis and correcting tumor cell drug resistance, and eventually improving quality of life (QoL) [12, 13]. To many oncology patients, TCM has become an option in the treatment of CRF due to its potential efficacy, but the potential of adverse reactions and toxicity should also be taken into consideration [14].

Recently, a growing body of clinical trials is emerging to investigate the efficacy and safety of TCM in treating CRF. A systematic review in 2014 reported that combining Chinese herbal medicines with chemotherapy or supportive care was more beneficial to improving QoL of CRF patients when compared with the treatment using chemotherapy or supportive care alone [10]. Another review, which analyzed 11 clinical trials that evaluated fatigue severity, QoL, activities of daily life and incidence of adverse events among lung cancer patients, found that, as compared to the use of conventional medicines only, combining herbal medicines with conventional medicines showed additional effectiveness and safety for CRF [15]. However, the number of the randomized controlled trial (RCTs) considered in these systematic reviews is still limited. In order to improve the evidence-base about using TCM for CRF, it is necessary to critically assess the emerging evidence from the RCTs. Therefore, this study aims to evaluate the efficacy and safety of TCM reported in RCTs. It is anticipated that the findings will be useful to inform the management of CRF for the patients and clinicians.

## Methods

This study was a systematic literature review conducted and reported in compliance with the updated Preferred Reporting Items for Systematic Reviews and Meta analyses (PRISMA) guidelines [16]. The Consolidated Standards of Reporting Trials Statement extensions for Chinese herbal medicine Formulas (CONSORT-CHM) [17] and the Cochrane Collaboration’s Risk of Bias tool [18] were used in this review to evaluate the reporting quality and the risk of bias of all included trials. The protocol of this systematic review has been registered on PROSPERO (CRD42023413625). [https://www.crd.york.ac.uk/prospero/display_record.php?ID=CRD42023413625].

### Types of studies

Randomized controlled trials which investigated the use of TCM in CRF regardless of blinding, status, date or language of publication were considered eligible for this review. Open-label experiment and observational studies were excluded.

### Types of RCTs participants

Participants of any age, gender or ethnic origin with a diagnosis of CRF, or the presence of fatigue after chemotherapy were eligible for the studies. RCTs in which participants had other diseases resulting in fatigue symptoms, or non-drugs treatments were excluded.

### Types of interventions

The interventions in the included RCTs for the treatment of CRF were divided into two categories, primary interventions and behavioral interventions. In particular, the primary interventions usually included TCM, western medicine (WM), TCM plus western medicine (TCM + WM), placebo or no treatment; behavioral interventions were set for chemotherapy (CH), other treatment (OT) and chemotherapy plus other treatment (CH + OT). Other treatment (OT) referred to the conventional treatment, symptomatic treatment, supportive treatment, nutritional therapy or other non-drug treatment for cancer patients after chemotherapy.

Primary interventions:

- TCM
- Western medicine (WM)
- TCM plus western medicine (TCM + WM)
- Placebo
- No treatment

Behavioral interventions:

- Chemotherapy (CH)
- Other treatment (OT)
- Chemotherapy plus other treatment (CH + OT)

### Types of outcomes

Both efficacy and safety of the TCM investigated in the included RCTs were analyzed. As such, the primary and the secondary outcomes of managing CRF with the use of TCM were as follows:

#### Primary outcomes

The primary outcome measures under consideration included changes in Piper Fatigue Scale (PFS) and the scales of QoL. On the one hand, PFS consists of twenty-two items and four subscales in total, six items of behavioral/severity, five items of affective meaning, five items of sensory and six items of cognitive/mood [19, 20]. Each item is scored from 0 (no fatigue) to 10 (most severe fatigue). Higher total scores indicate more severe fatigue.

On the other hand, QoL is defined as: “an individual's perception of their position in life in the context of the culture and value systems in which they live and in relation to their goals, expectations, standards and concerns” from the World Health Organization (WHO) [21, 22]. As a significant indicator in medical and health research, QoL is mainly applied to patients with specific cancer and long-term diseases [23]. For example, the scales of KPS (Karnofsky Performance Status), QLQ-C30 (EORTC Quality of Life Questionnaires), GQoLI-74 (Generic Quality of Life Inventory-74), SF-36 (36-item Short-Form), FACT-F (Functional Assessment of Cancer Therapy–Fatigue), FACT-G (Functional Assessment of Cancer Therapy–General), FACIT-F (Functional Assessment of Chronic Illness Therapy–Fatigue), FACT-ES (Functional Assessment of Chronic illness Therapy–Endocrine Symptoms), PSQI (Pittsburgh sleep quality index), QoL^1^ (Quality of Life Questionnaire); QoL^2^ (Quality of Life Questionnaire and Quality of life score (QoL) for cancer patients (Chinese version draft) were used to evaluate the QoL when TCM was used for treating CRF in this review.

#### Secondary outcomes

The secondary outcome measures were TCM syndrome score, fatigue scales, cytokines tests, blood tests, other scales and indicators:

1. TCM syndrome score: According to the Chinese Medicine Clinical Research of New Drugs Guiding Principles [11], TCM syndrome scores are mainly used to evaluate symptoms such as fatigue, shortness of breath, lazy speech, irritability, insomnia, and weakness of the waist and knees, divided into three levels, for mild ( +), moderate (+ +), and severe (+ + +), and the relative scores are 1 point, 2 points, and 3 points respectively. The higher the score, the more severe the symptoms.
2. Fatigue scales: BFI (Brief Fatigue Inventory), CFS (Cancer Fatigue Scale), CFS* (Chalder Fatigue Scale), MFSI-SF (Multidimensional Fatigue Symptom Inventory–Short Form), FSI (Fatigue Symptom Inventory), BFI-C (Chinese Version of Brief Fatigue Inventory), TOI-F (Trial Outcome Index–Fatigue), VAS-F (Visual Analogue Scale of Global Fatigue), NGFRS (NCCN Guidelines Fatigue Rating Scale)
3. Other scales: HADS (Hospital Anxiety and Depression Scale), POMS (Profile of Mood States), HAMA (Hamilton Anxiety Scale), SDS (Self-Rating Depression Scale), SS (satisfaction survey), CS (Comfort Survey), PSS (Perceived stress scale), GIC (the Global Impression of Change), LASA (Linear Analogue Self Assessment Scale), BDI (Beck Inventory Depression Scale), GCSG (Gastric Cancer Symptom Grading Scale), LCSG (Lung Cancer Symptom Grading Scale)
4. Cytokines tests: CD3^+^, CD4^+^, CD8^+^, CD3^+^CD4^+^, CD3^+^CD8^+^, CD4^+^/CD8^+^, CD16^+^56^+^, TNF-α, IFN-γ, IL-1, IL-1β, IL-2, IL-4, IL-5, IL-6, IL-8, IL-10, NK cell, IgG, IgA, IgM, TGF-β.
5. Blood tests: hemoglobin (HGB), albumin (Alb), white blood cell (WBC), total protein (TP), platelet (PLT), cortisol (COR), red blood cells (RBC), mean corpuscular hemoglobin (MCH), mean corpuscular hemoglobin concentration (MCHC), mean corpuscular volume (MCV), hematocrit (HCT), degree of myelosuppression (DM)
6. Others: weight, cardiac function, liver function, kidney function, blood viscosity, urinalysis, Recombinant Human Granulocyte Colony Stimulating Factor (rhG-CSF) dosage, physical and chemical indicators, WHO Response Evaluation Criteria in Solid Tumors (RECIST), improve cancer cachexia-related symptoms, standards for the diagnosis and treatment of common malignant tumors in China.

### Search strategies

This systematic review was conducted according to the PRISMA literature research guidelines [24]. Seven electronic databases including PubMed, the Cochrane Library, Embase, Web of Science (WOS), Scopus, China National Knowledge Infrastructure (CNKI) and Wanfang database were considered to identify RCTs which evaluated TCM in treating CRF from database inception to December 2022. The three primary search terms were “CRF”, “TCM” and “RCTs”.

As shown in Table 1, to ensure an effective search, Medical Subject Headings (MeSH) terms were used to develop a comprehensive search strategy. Common phrases and keywords related to the three terms (CRF, TCM, RCTs) were combined with OR. The results from each concept were combined with AND. A detailed description of each search strategy is provided in Additional file 1.

**Table 1:** Search term identifiers

Category	Entry search terms in English	Entry search terms in Chinese
1. Cancer-related fatigue (CRF)	“Fatigue” [Mesh]	癌因性疲勞
	“Lassitude”	癌因性疲乏
		癌性疲勞
		癌性疲乏
		癌相關性疲勞
		腫瘤相關性疲勞
		腫瘤相關性疲乏
2. Traditional Chinese medicine (TCM)	“Medicine, Traditional” [Mesh]	中藥
	“Complementary Therapies” [Mesh]	中醫藥
	“Phytotherapy” [Mesh]	中草藥
	“Plant Extracts” [Mesh]	
	“Plants, Medicinal” [Mesh]	
	“Drugs, Chinese Herbal” [Mesh]	
	“Medicine, Chinese Traditional” [Mesh]	
	“Complementary medicine*”	
	“Alternative medicine*”	
	“Chinese medicine*”	
	“Pharmaceutical plant*”	
	“Medicinal plant*”	
	“Herb*”	
	“Nutraceutical*”	
	“Folk remedy*”	
	“Folk medicine*”	
3. Randomized controlled trials (RCTs)	Randomized clinical trial*	隨機 + 對照 + 隨機對照

“ + ” retrieved results that included all the search terms

^*^Including but not limited to

### Exclusion criteria and screening

This review only included studies that reported the results of RCTs investigating the efficacy and/or safety of TCM in CRF. Studies subjected to exclusion were: (1) review, meta-analysis, protocol; (2) non-randomized trial; (3) pharmacodynamics or pharmacology studies; (4) animal experiments; (5) studies on other non-drugs therapy (such as acupuncture, qigong, music, and behavior); (6) studies on other diseases or fatigue caused by other diseases.

### Data extraction and analysis

All references were categorized and archived in Endnote X9, data was recorded and organized using the EXCEL 2013. As listed in Table 2, relevant data from all eligible studies were extracted according to a standard extraction form, which included basic information of studies, methods, interventions, participants, outcomes, and overall findings.

**Table 2:** Relevant data from all included literature extracted for further analysis

Data category	Items
1. Basic information of study	▪ Information on the first author ▪ Publication year
2. Methods	▪ Trial design ▪ Date and setting of the trial ▪ Criteria for inclusion and exclusion of patients ▪ Criteria for diagnosing patients
3. Intervention	▪ The TCM and its dosage used in the experimental group ▪ The comparator in the control groups ▪ Intervention duration
4. Participants	▪ The number of participants in the randomization phase ▪ The number of participants in the analysis phase ▪ Mean age of patients ▪ The sex ratio ▪ History of CRF ▪ Dropouts
5. Outcomes and overall findings	▪ Primary outcomes, secondary outcomes, and other outcomes ▪ Difference of efficacy rate between two group ▪ Safety assessed either quantitatively or qualitatively in terms of adverse effects reported

### Assessment of reporting quality

Two of the authors (JY, YL) independently assessed the included eligible studies based on 25-item of CONSORT-CHM 2017 statement [17]. This statement provided a grading system devised for each criterion that was used to determine the quality of every clinical trial of TCM interventions. According to the degree of conformity, the assessment results for each item were determined as non-existent, partially present and fully compliant.

### Assessment of risk of bias

Cochrane Collaboration’s Risk of Bias [18] tool was used to evaluate the quality of each studies by two authors (JY, YL). The judgment was based on the definition of the recommendation by the Cochrane Handbook for Systematic Reviews of Interventions, and the assessment results for each item were grouped into one of the following three categories: “low risk of bias”, “unclear risk of bias”, and “high risk of bias”. Further explanation about the risk assessment was shown in Additional file 2: Table S1.

## Results

### Search results

**Fig. 1:**
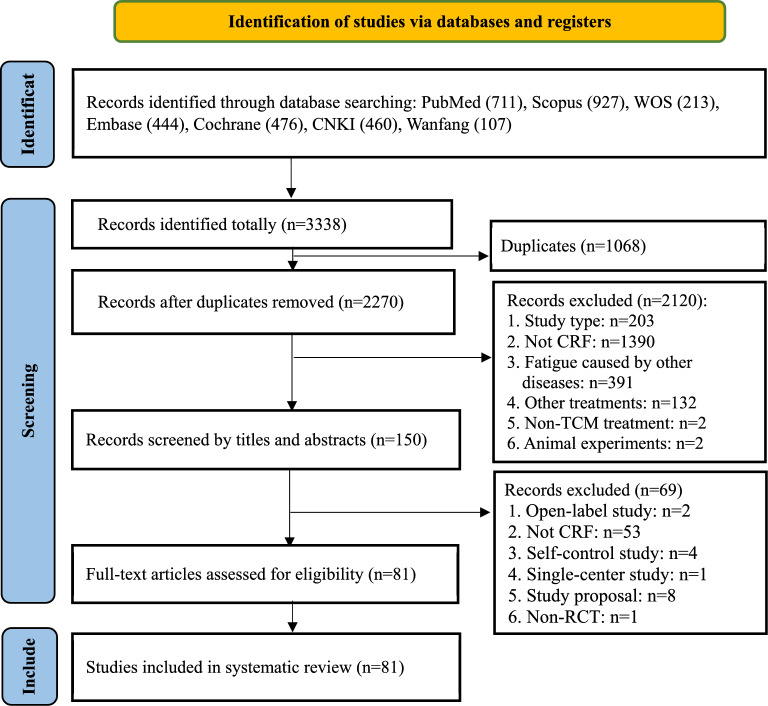
PRISMA flow-chart of study selection

The screening process conducted in accordance with the PRISMA guidelines is summarized in the flow diagram as shown in Fig. 1. A total of 3338 records were initially yielded from the seven electronic databases and related sources. After removing 1068 duplicates, 2270 relevant articles were retained for screening. After title and abstract screening, 2120 records were excluded due to various reasons: study or publication type (review or meta-analysis or protocol articles or others (n = 203)), focus on conditions other than CRF (n = 1390), focus on symptoms of fatigue developed from other diseases (n = 391), focus on other treatments such as food, acupuncture, moxibustion or others (n = 132), non-TCM treatment (n = 2), and animal trials (n = 2). A total of 150 articles were retained for the subsequent full-text screening and 69 records were further excluded due to the following reasons: open-label study (n = 2), focus on conditions other than CRF (n = 53), self-control study (n = 4), single-center study (n = 1), study proposal for CRF (n = 8) and non-RCT (n = 1). Eventually, 81 eligible articles published in Chinese databases (n = 70) or English databases (n = 11) were included in this review.

### Description of studies

Since one of the included studies reported two RCTs [25], this systematic review comprised 82 trials from 81 publications. In particular, 71 studies were published in Chinese and only 10 studies were published in English [25–34]. Except for some trials conducted in Korea (n = 3) [28, 33, 34], Brazil (n = 3) [25, 29], USA (n = 2) [27, 31], France (n = 1) [26], Italy(n = 1) [32], the remaining 72 trials were conducted in China. More details about the studies are shown in Table 3.

**Table 3:** Basic descriptions of the studies included in this review

No.	Author, year	Name of TCM preparation	Major ingredients	Form of preparation	Cancer classification	Study period	Number of participants (test group, control group)	Number of male and female	Participants’ mean age (years)
1	(Gu et al. 2021) [35]	Buzhong Yishen Decoction	Astragali Radix, Psoraleae Fructus, Glycyrrhizae Radix, Epimedii Folium, Cuscuta chinensis Lam., Hedyotis diffusa Willd., Lycii Fructus, Bupleuri Radix, Atractylodis Macrocephalae Rhizoma, Citri Reticulatae Pericarpium, Codonopsis Radix, Pinelliae Rhizoma, Cimicifugae Rhizoma, Scutellariae Radix, Angelicae Sinensis Radix	Decoction	Stomach:36; colon:24	2019.1–2020.3	60 (30, 30)	M = 40, F = 20	T: 62.53 ± 5.84 C: 63.07 ± 5.90
2	(Li et al. 2020) [36]	Shenqi Fuzheng Injection	Codonopsis Radix, Astragali Radix	Injection	Lung:55; stomach:5	2016.4–2017.2	60 (31, 29)	M = 36, F = 24	T: 65.52 ± 11.01 C: 61.66 ± 11.27
3	(Lin et al. 2020) [37]	Xiaopi Decoction	Astragali Radix, Ginseng Radix, Glycyrrhizae Radix, Angelicae Sinensis Radix, Citri Reticulatae Pericarpium, Atractylodis Macrocephalae Rhizoma, Cimicifugae Rhizoma, Bupleuri Radix, Angelicae Sinensis Radix, Rehmanniae Radix, Eucommiae Cortex, Lycii Fructus, Polygoni Multiflori Radix	Decoction	Others:45	2017.1–2018.12	45 (23, 22)	M = 29, F = 16	T: range: 30–83 C: range: 39–82
4	(Hu et al. 2020) [38]	Buzhong Yiqi Decoction	Zingiber officinale Roscoe, Codonopsis Radix, Ziziphus jujuba Mill, Atractylodis Macrocephalae Rhizoma, Euryale ferox Salisb, Angelicae Sinensis Radix, Astragali Radix	Decoction	Breast:72	2018.12–2019.12	72 (36, 36)	N/A	T: 54.23 ± 5.70 C: 54.56 ± 5.65
5	(Wang et al. 2016) [39]	Yiqi Jianpi Decoction	Codonopsis Radix, Atractylodis Macrocephalae Rhizoma, Poria, Glycyrrhizae Radix, Astragali Radix, Dioscoreae Rhizoma, Angelicae Sinensis Radix, Corni Fructus, Lycii Fructus, Rehmanniae Radix, Citri Reticulatae Pericarpium, Vatica mangachapoi Blauco, Pinelliae Rhizoma, Crataegi Fructus, Massa Medicata Fermentata et.al	Decoction	Breast:75	2010.1–2014.10	75 (38, 37)	M = 0, F = 75	range: 22–83 mean: 57
6	(Zhao et al. 2011) [30]	Spore powder of Ganoderma Lucidum	G. lucidum	Powers	Breast:48	2009.6–2010.9	48 (25, 23)	M = 0, F = 48	T: 51.3 ± 9.8 C: 53.2 ± 8.7
7	(Cao, 2020) [40]	Jiawei Sijunzi Recipe	Codonopsis Radix, Atractylodis Macrocephalae Rhizoma, Poria, Glycyrrhizae Radix, Colla Carapacis et Plastri Testudinis, Lycii Fructus, Epimedii Folium	Decoction	Lung:6; breast:13; stomach:11; ovarian:8; colorectum:11; ureteral:2; diffuse large B cell lymphoma:9	N/A	60 (30, 30)	M = 32, F = 28	T: 64.8 ± 4.6 C: 64.5 ± 4.3
8	(Ou et al. 2022) [41]	Jianpi Yangrong Decoction	Ginseng Radix et Rhizoma, Poria, Atractylodis Macrocephalae Rhizoma; Glycyrrhizae Radix, Paeoniae Radix Alba, Chuanxiong Rhizoma, Rehmanniae Radix, Angelicae Sinensis Radix, Astragali Radix, Polygonati Rhizoma, Dioscoreae Rhizoma	Decoction	Liver:60	2020.6.1–2021.6.1	60 (30, 30)	M = 42, F = 18	T: 48.92 ± 10.12 C: 50.27 ± 11.16
9	(Zhang et al. 2018) [42]	Zini Sizibuxu Decoction	Psoraleae Fructus, Zingiberis Rhizoma, Glycyrrhizae Radix, Astragali Radix, Amomi Fructus, Galli Gigeriae Endothelium Corneum, Aucklandiae Radix, Codonopsis Radix, Poria, Atractylodis Macrocephalae Rhizoma, et.al	Decoction	Lung:21; breast:13; stomach:8; ovarian:4; bowel:10	N/A	56 (28,28)	M = 30, F = 26	range: 45–76
10	(Zhu et al. 2016) [43]	Buzhong Yiqi Decoction	Astragali Radix, Codonopsis Radix, Angelicae Sinensis Radix, Citri Reticulatae Pericarpium, Cimicifugae Rhizoma, Bupleuri Radix, Atractylodis Macrocephalae Rhizoma, Glycyrrhizae Radix	Decoction	Stomach:60	N/A	60 (34, 26)	N/A	N/A
11	(Wang et al. 2015) [44]	Yiqi Jianpi Recipe	Codonopsis Radix, Atractylodis Macrocephalae Rhizoma, Poria, Glycyrrhizae Radix, Coicis Semen, Dioscoreae Rhizoma, Citri Reticulatae Pericarpium, Fructus oryzae germinatus, Fructus Hordei Germinatus, Galli Gigeriae Endothelium Corneum, et.al	Decoction	Lung:124	2010.7–2012.2	124 (63, 61)	M = 76, F = 48	T: 58.71 ± 10.20 C: 57.41 ± 10.33
12	(Chen, 2011) [45]	Buzhong Yiqi Decoction	Astragali Radix, Atractylodis Macrocephalae Rhizoma, Citri Reticulatae Pericarpium, Cimicifugae Rhizoma, Bupleuri Radix, Codonopsis Radix, Glycyrrhizae Radix, Angelicae Sinensis Radix	Decoction	Breast:60	2010.2–2011.5	60 (30, 30)	M = 0, F = 60	range: 27–70
13	(Yang et al. 2015) [46]	Shuyu Pill	Dioscoreae Rhizoma, Angelicae Sinensis Radix, Ramulus Cinnamomi, Massa Medicata Fermentata, Rehmanniae Radix, Sojae Semen Germinatum, Glycyrrhizae Radix, Ginseng Radix, Asini Corii Colla, Chuanxiong Rhizoma, Paeoniae Radix Alba, Atractylodis Macrocephalae Rhizoma, Ophiopogonis Radix, Saposhnikoviae Radix, Armeniacae Semen Amarum, Bupleuri Radix, Platycodonis Radix, Poria, Zingiberis Rhizoma, Ampelopsis Radix, Ziziphus jujuba Mill	Pills	Lung:23; breast:17; stomach:12; ovarian:11; bowel:13; pancreatic:10	2011.6–2013.11	86 (43, 43)	M = 42, F = 44	T: range: 35–69 C: range: 33–67
14	(Kong et al. 2016) [47]	Fuzheng Yiliu Decoction	Astragali Radix, Codonopsis Radix, Atractylodis Macrocephalae Rhizoma, Poria, Aurantii Fructus, Magnoliae Officinalis Cortex, Armeniaca mume Sieb. var. mume f. viridicalyx (Makino) T. Y. Chen, Scutellaria barbataD. Don, Polygoni Cuspidati Rhizoma et Radix, Coicis Semen, Hedyotis diffusa Willd., Amomi Fructus, Fructus oryzae germinatus	Decoction	Lung:60	2013.8–2015.11	60 (32, 28)	M = 38, F = 22	T: 58.67 ± 10.39 C: 59.37 ± 11.78
15	(Su et al. 2022) [48]	Chaiqi Sanhua Decoction	Astragali Radix, Bupleuri Radix, Rosa rugosa Thunb, Armeniaca mume Sieb, Albizia julibrissin Durazz, Cimicifugae Rhizoma, Glycyrrhizae Radix, Codonopsis Radix, Atractylodis Macrocephalae Rhizoma, Paeoniae Radix Alba, Citrus junos Sieb. ex Tanaka, Angelicae Sinensis Radix, Poria	Decoction	Breast:56	2019.6–2021.12	56 (28, 28)	M = 2, F = 54	T: 52.86 ± 9.04 C: 52.21 ± 9.55
16	(Jiang, 2022) [49]	Kangai Injection	Ginseng Radix, Astragali Radix, Sophorae Flavescentis Radix	Injection	Breast:17; esophageal:24; bowel:19	2019.11–2021.5	60 (30, 30)	M = 35, F = 25	T: 55.76 ± 2.37 C: 55.33 ± 2.45
17	(Luo et al. 2021) [50]	Zini Zhongyao Decipe	Pseudostellariae Radix, Poria, Dioscoreae Rhizoma, Dolicho LablabL, Coicis Semen, Crataegi Fructus, Massa Medicata Fermentata, Galli Gigeriae Endothelium Corneum, Fritillariae Thunbergii Bulbus, Fruit of austral Akenia, Atractylodis Macrocephalae Rhizoma, Platycodonis Radix, Citri Reticulatae Pericarpium, Hive, Scolopendra, Amomi Fructus	Decoction	Lung:42; breast:5; stomach:17; bowel:14; kidney:2; gynecological:4	2018.1–2018.11	84 (43, 41)	M = 42, F = 42	T: 63.58 ± 10.14 C: 67.26 ± 10.01
18	(Wang et al. 2021) [51]	Kangai Injection	Ginseng Radix, Astragali Radix, Sophorae Flavescentis Radix	Injection	Stomach:5; liver:21; esophageal:2; colon:4; pancreatic:6; gallbladder:2	2017.12–2020.6	40 (20, 20)	M = 25, F = 15	range: 33–83; mean: 62 ± 5.4
19	(He et al. 2020) [52]	Zini Jianpi Bushen Decipe	Pseudostellariae Radix, Atractylodis Macrocephalae Rhizoma, Poria, Aucklandiae Radix, Amomi Fructus, Dolicho LablabL, Dioscoreae Rhizoma, Semen Nelumbinis Taxilli Herba, Dipsaci Radix, Eucommiae Cortex, Portulaca oleracea L, Thlaspi arvense Linn, Sophora japonica Linn, Sanguisorba officinalis L, Glycyrrhizae Radix	Decoction	Bowel:68	2018.1–2018.12	68 (34, 34)	M = 37, F = 31	T: 59.97 ± 9.64 C: 62.56 ± 7.55
20	(Luo et al. 2019) [53]	Shenqi Fuzheng Injection	Codonopsis Radix, Astragali Radix	Injection	Lung:78; digestive tract:115	2017.11–2018.9	193 (107, 86)	M = 111, F = 82	T: 60.3 ± 6.5 C: 61.2 ± 3.4
21	(Liu, 2018) [54]	Guipi Decoction	Atractylodis Macrocephalae Rhizoma, Angelicae Sinensis Radix, Poria, Astragali Radix, Polygalae Radix, Arillus Loongan, Ziziphi Spinosae Semen, Ginseng Radix, Aucklandiae Radix, Glycyrrhizae Radix	Decoction	Digestive tract:64	2014.1–2018.6	64 (32, 32)	N/A	N/A
22	(Lin et al. 2018) [55]	Buzhong Yiqi Decoction	Astragali Radix, Codonopsis Radix, Atractylodis Macrocephalae Rhizoma, Glycyrrhizae Radix, Angelicae Sinensis Radix, Citri Reticulatae Pericarpium, Cimicifugae Rhizoma, Bupleuri Radix, Zingiber officinale Roscoe, Ziziphus jujuba Mill	Decoction	Lung:64	2016.9–2017.9	64 (32, 32)	M = 37, F = 27	T: range: 55–74; mean: 64.8 C: range: 54–76, mean: 64.5
23	(Li, 2016) [56]	Jianpi Yishen Decoction	Codonopsis Radix, Astragali Radix, Atractylodis Macrocephalae Rhizoma, Poria, Glycyrrhizae Radix, Ligustri Lucidi Fructus, Lycii Fructus, Corni Fructus, Psoraleae Fructus	Decoction	Colorectum:60	2013.5–2015.1	60 (30, 30)	M = 29, F = 31	T: 69.50 ± 8.03 C: 68.13 ± 6.77
24	(Song et al. 2016) [57]	Yangzheng Xiaoji Capsule	Ginseng Radix, Atractylodis Macrocephalae Rhizoma, Poria, Curcumae Rhizoma, Galli Gigeriae Endothelium Corneum, Eupolyphaga, Gynostemma pentaphyllum (Thunb.) Makino, Hedyotis diffusa Willd., Scutellaria barbataD. Don, Solanum lyratum Thunb, Duchesnea indica (Andr.) Focke, Cynanchum paniculatum (Bunge) Kitagawa	Capsules	*Lung:23; breast:21; liver:20; cervical:5; rectal:15	2013.1–2015.6	90 (45, 45)	*M = 46, *F = 38	*T: 41.7 ± 11.4 *C: 40.2 ± 11.7
25	(Liu et al. 2016) [58]	Buzhong Yiqi Decoction	Codonopsis Radix, Astragali Radix, Angelicae Sinensis Radix, Lycii Fructus, Euryale ferox Salisb, Atractylodis Macrocephalae Rhizoma, Zingiber officinale Roscoe, Ziziphus jujuba Mill	Decoction	Lung:20; breast:24; rectal:20; colon:18	2014.5–2015.12	82 (41, 41)	M = 45, F = 37	< 65: 40 ≥ 65: 42
26	(Li 2016) [59]	Jianpi Yishen Recipe	Codonopsis Radix, Atractylodis Macrocephalae Rhizoma, Poria, Glycyrrhizae Radix, Coicis Semen, Pinelliae Rhizoma, Corni Fructus, Psoraleae Fructus, Citri Reticulatae Pericarpium, et.al	Decoction	Bowel:150	2014.1–2015.1	150 (75, 75)	M = 88, F = 62	T: 52.98 ± 5.71 C: 54.21 ± 4.93
27	(Zhang et al. 2016) [60]	Shenqi Fuzheng Injection	Codonopsis Radix, Astragali Radix	Injection	Lung:52	2014.9–2015.3	52 (26, 26)	M = 25, F = 27	T: 61.35 ± 6.71 C: 61.46 ± 6.78
28	(Liang et al. 2016) [61]	Shengxuebao Mixture	Polygoni Multiflori Radix, Ligustri Lucidi Fructus, Fructus Mori, Eclipta Prostrata L., Paeoniae Radix Alba, Astragali Radix, Cibotii Rhizoma	Oral solutions	Lung:10; breast:12; cervical:12; colorectum:14; nasopharyngeal carcinoma:12	N/A	60 (30, 30)	M = 29, F = 31	T: range: 32–69; median: 51.3 C: range: 34–73, median: 54.5
29	(Sun 2015) [62]	Kangai Injection	Ginseng Radix, Astragali Radix, Sophorae Flavescentis Radix	Injection	Stomach:38; esophageal:29; colorectum:36	2012.9–2014.10	103 (60, 43)	*M = 64, F = 38	range: 36–80; median: 62
30	(Li et al. 2011) [63]	Shenfu Injection	Ginseng Radix, Aconiti Lateralis Radix Praeparata	Injection	Lung:182	2008.4–2011.3	182 (62, 62, 58)*	M = 112, F = 70	55.39 ± 7.98
31	(Huang 2001) [64]	Kanglai te Injection	Coicis Semen	Injection	Lung:9; breast:8; stomach:21; liver:12; ovarian:2; cervical:1; esophageal:4; colorectum:12; laryngeal: 1; bladder: 1; prostate: 2; malignant lymphoma: 2; nasopharyngeal carcinoma: 3	1994.12–1998.12	78 (36, 42)	M = 54, F = 24	T: range: 32–75, mean: 54.6 C: range: 33–78, mean: 55.6
32	(Cao et al. 2022) [65]	Sini plus Renshen Decoction	Aconitum carmichaeli Debx., Zingiberis Rhizoma, Ginseng Radix, Glycyrrhizae Radix	Decoction	Lung:25; breast:13; stomach:4; bowel:19; kidney:3; nasopharyngeal carcinoma:4	2017.3–2019.12	68 (34, 34)	M = 37, F = 31	T: 50.69 ± 8.24 C: 50.58 ± 8.15
33	(Zhang et al. 2019) [66]	Jiawei Sijunzi Decoction	Pseudostellariae Radix, Atractylodis Macrocephalae Rhizoma, Fructus Mori, Polygonati Rhizoma, Ziziphus jujuba Mill, Dioscoreae Rhizoma, Poria	Decoction	Others:60	2016.1.18–2017.7.18	60 (30, 30)	M = 35, F = 25	T: 47.77 ± 8.48 C: 46.10 ± 8.18
34	(Wang, 2019) [67]	Buzhong Yiqi Decoction	Euryale ferox Salisb, Atractylodis Macrocephalae Rhizoma, Lycii Fructus, Angelicae Sinensis Radix, Zingiber officinale Roscoe, Astragali Radix, Codonopsis Radix, Ziziphus jujuba Mill	Decoction	Breast:80	2016.4–2018.2	80 (40, 40)	N/A	T: 57.3 ± 5.8 C: 56.2 ± 5.6
35	(Yao, 2019) [68]	Shengxian Decoction	Astragali Radix, Ziziphus Jujuba Mill, Bupleuri Radix, Cimicifugae Rhizoma, Anemarrhenae Rhizoma, Platycodonis Radix, Glycyrrhizae Radix, et.al	Decoction	Lung:71	2016.1–2019.3	71 (36, 35)	M = 44, F = 27	T: 68.59 ± 7.4 C: 68.53 ± 6.5
36	(Chen et al. 2019) [69]	Bazhen Decoction + Shenqi Fuzheng Injection	Codonopsis Radix, Poria, Rehmanniae Radix, Paeoniae Radix Alba, Angelicae Sinensis Radix, Atractylodis Macrocephalae Rhizoma, Chuanxiong Rhizoma, Glycyrrhizae Radix, et.al	Decoction and Injections	Stomach:60	2013.12–2016.9	60 (30, 30)	M = 30, F = 30	T: 52.13 ± 4.17 C: 52.13 ± 4.17
37	(Wang, 2018) [70]	Shenqi Fuzheng Injection	Codonopsis Radix, Astragali Radix	Injection	Stomach:40	2015.6–2017.12	40 (20, 20)	M = 25, F = 15	T: 65.9 ± 10.198 C: 64.4 ± 12.713
38	(Guo et al. 2017) [71]	Shenqi Fuzheng Injection	Codonopsis Radix, Astragali Radix	Injection	Lung:89	2014.9–2016.10	89 (44, 45)	M = 55, F = 34	T: 64.5 ± 5.69 C: 62.9 ± 6.17
39	(Gu et al. 2009) [72]	Shenqi Fuzheng Injection	Codonopsis Radix, Astragali Radix	Injection	Breast:13; stomach:26; rectal:15; colon:22	2004.10–2007.10	76 (40, 36)	M = 38, F = 38	T: range: 33–70, mean: 53.7 C: range: 31–73, mean: 54.3
40	(Liu et al. 2014) [73]	Zini Yiqiyangxue Recipe	Notoginseng Radix, Fructus Hordei Germinatus, Massa Medicata Fermentata, Crataegi Fructus, Pinelliae Rhizoma, Citri Reticulatae Pericarpium, Amomi Fructus, Rehmanniae Radix, Asini Corii Colla, Dried Human Placenta, Tortoise Plastron, Angelicae Sinensis Radix, Codonopsis Radix, Astragali Radix	Decoction	Colorectum:50	2010–2012	50 (25, 25)	M = 28, F = 22	T: 61.4 ± 7.25 C: 61.7 ± 9.05
41	(Li et al. 2013) [74]	Fufang Ejiao Jiang	Asini Corii Colla, Ginseng Radix, Rehmanniae Radix, Codonopsis Radix, Crataegi Fructus	Oral solutions	Lung:23; breast:11; stomach:5; ovarian:11; bowel:13; kidney:5; nasopharyngeal carcinoma:5	2010.5–2012.1	73 (37, 36)	M = 42, F = 31	T: 61 ± 11 C: 60 ± 10
42	(Wang et al. 2016) [75]	Guishao Liujunzi Decoction	Codonopsis Radix, Atractylodis Macrocephalae Rhizoma, Poria, Citri Reticulatae Pericarpium, Pinelliae Rhizoma, Glycyrrhizae Radix, Angelicae Sinensis Radix, Paeoniae Radix Alba, et.al	Decoction	Colorectum:60	2013.1–2015.10	60 (30, 30)	M = 35, F = 25	T: range: 38–78, mean: 62 C: range: 34–75, mean: 59
43	(Cai 2016) [76]	Buzhong Yiqi Decoction	Astragali Radix, Citri Reticulatae Pericarpium, Bupleuri Radix, Angelicae Sinensis Radix, Atractylodis Macrocephalae Rhizoma, Cimicifugae Rhizoma, Codonopsis Radix	Decoction	Lung:13; breast:14; stomach:27; liver:14; esophageal:24; bowel:8; pancreatic:2; gallbladder: 6; nasopharyngeal carcinoma: 2	2014.1–2016.1	110 (55, 55)	M = 50, F = 60	T: range: 46–84 C: range: 45–85
44	(Ning et al. 2020) [77]	Jiawei Buzhonyiqi Decoction	Astragali Radix, Codonopsis Radix, Atractylodis Macrocephalae Rhizoma, Cimicifugae Rhizoma, Bupleuri Radix, Angelicae Sinensis Radix, Citri Reticulatae Pericarpium, Pinelliae Rhizoma, Ophiopogonis Radix, Schisandrae Chinensis Fructus, Glycyrrhizae Radix	Decoction	Lung:80	2018.10–2019.10	80 (40, 40)	*M = 43, F = 30	*T: 60.5 ± 11.8 C: 59.9 ± 9.3
45	(Yang 2020) [78]	Kangai Injection	Ginseng Radix, Astragali Radix, Sophorae Flavescentis Radix	Injection	Lung:82	2018.2–2019.10	82 (41, 41)	M = 48, F = 34	T: 58.01 ± 5.97 C: 57.99 ± 6.12
46	(Shan et al. 2020) [79]	Kangai Injection	Ginseng Radix, Astragali Radix, Sophorae Flavescentis Radix	Injection	Lung:90	2017.8–2019.7	90 (45, 45)	M = 51, F = 39	T: 58.60 ± 4.21 C: 58.30 ± 4.44
47	(Wu 2018) [80]	Kangai Injection	Ginseng Radix, Astragali Radix, Sophorae Flavescentis Radix	Injection	Lung:54	2016.2–2017.2	54 (27, 27)	M = 35, F = 19	T: 57.33 ± 6.21 C: 4.36 ± 1.23
48	(Shi 2017) [81]	Kangai Injection	Ginseng Radix, Astragali Radix, Sophorae Flavescentis Radix	Injection	Lung:90	2015.3–2016.3	90 (45, 45)	M = 51, F = 39	T: 62.5 ± 5.0 C: 62.0 ± 4.8
49	(Zhang et al. 2017) [82]	Kangai Injection	Ginseng Radix, Astragali Radix, Sophorae Flavescentis Radix	Injection	Lung:96	2015.1–2015.12	96 (48, 48)	M = 63, F = 33	T: 52.12 ± 2.16 C: 51.43 ± 2.13
50	(Wang et al. 2015) [83]	Kangai Injection	Ginseng Radix, Astragali Radix, Sophorae Flavescentis Radix	Injection	Lung:70	2012.9–2014.5	70 (35, 35)	M = 44, F = 26	T: range: 46–70, mean: 52.94 C: range:43–70, mean: 53
51	(Lu 2014) [84]	Kangai Injection	Ginseng Radix, Astragali Radix, Sophorae Flavescentis Radix	Injection	Lung:33; breast:23; stomach:24; colorectum:38; others:15	2012.5–2013.9	133 (68, 65)	M = 69, F = 64	T: range: 37–82, mean: 60 C: range: 39–80, mean: 59
52	(Zhang et al. 2019) [85]	Guipi Decoction	Atractylodis Macrocephalae Rhizoma, Angelicae Sinensis Radix, Poria, Astragali Radix, Polygalae Radix, Arillus Loongan, Ziziphi Spinosae Semen, Ginseng Radix, Aucklandiae Radix, Glycyrrhizae Radix	Decoction	Gastrointestinal: 68	2018.3–2019.4	68 (34, 34)	M = 35, F = 33	T: 55.30 ± 12.11 C: 55.18 ± 11.03
53	(Zhang et al. 2017) [86]	Shenqi Granule	Codonopsis Radix, Astragali Radix	Granules	Colorectum:118	2013.5–2016.2	118 (59, 59)	*M = 62, F = 52	*T: 54.85 ± 5.3 C: 55.14 ± 5.27
54	(Wu et al. 2014) [87]	Shenmai Injection	Ginseng Radix, Ophiopogonis Radix	Injection	Lung:94	2011.3–2013.6	94 (47, 47)	M = 49, F = 45	T: 52.8 ± 10.5 C: 53.2 ± 10.2
55	(Feng 2014) [88]	Shenfu Injection	Ginseng Radix, Aconiti Lateralis Radix Praeparata	Injection	Lung:63	2010.6–2014.6	63 (31, 32)	M = 44, F = 19	T: 49.5 ± 7.6 C: 48.3 ± 6.8
56	(Zhang et al. 2012) [89]	Kangai Injection	Ginseng Radix, Astragali Radix, Sophorae Flavescentis Radix	Injection	Pancreatic:28	2010.8–2012.4	28 (16, 12)	M = 17, F = 11	T: range: 37–67, mean: 51.95 C: range: 34–69, mean: 52.03
57	(Li 2015) [90]	Jianpi Yishen Recipe	Codonopsis Radix, Atractylodis Macrocephalae Rhizoma, Poria, Glycyrrhizae Radix, Coicis Semen, Pinelliae Rhizoma, Corni Fructus, Psoraleae Fructus, Citri Reticulatae Pericarpium	Decoction	Colorectum:60	2012.4–2014.12	60 (30, 30)	M = 29, F = 31	T: 61.3 ± 10.4 C: 57.9 ± 12.8
58	(Yang et al. 2018) [91]	Buzhong Yiqi Decoction	Astragali Radix, Atractylodis Macrocephalae Rhizoma, Glycyrrhizae Radix, Ginseng Radix, Citri Reticulatae Pericarpium, Cimicifugae Rhizoma, Bupleuri Radix, Angelicae Sinensis Radix	Decoction	Lung:21; stomach:19; esophageal:15; rectal:14; nasopharyngeal carcinoma:11	2014.1–2016.12	80 (40, 40)	M = 49, F = 31	T: 74.89 ± 10.18 C: 75.01 ± 10.61
59	(Ou et al. 2016) [92]	Kangai Injection	Ginseng Radix, Astragali Radix, Sophorae Flavescentis Radix	Injection	Lung:80	2014.6–2015.6	80 (40, 40)	M = 47, F = 33	T: 60.8 ± 7.2 C: 61.2 ± 7.6
60	(Zhao 2015) [93]	Kangai Injection	Ginseng Radix, Astragali Radix, Sophorae Flavescentis Radix	Injection	Lung:80	2010.7–2013.7	80 (40, 40)	M = 48, F = 32	T: 57.8 ± 4.9 C: 58.2 ± 4.7
61	(Leng 2015) [94]	Kangai Injection	Ginseng Radix, Astragali Radix, Sophorae Flavescentis Radix	Injection	Lung:60	2012.7–2013.7	60 (30, 30)	M = 33, F = 27	T: range: 40–70 C: range: 45–75
62	(Jing et al. 2010) [95]	Kangai Injection	Ginseng Radix, Astragali Radix, Sophorae Flavescentis Radix	Injection	Lung:87	2007-	87 (44, 43)	M = 47, F = 40	T: range: 46–70, mean: 60.5 C: range: 45–70, mean: 61.3
63	(Huang 2012) [96]	Kangai Injection	Ginseng Radix, Astragali Radix, Sophorae Flavescentis Radix	Injection	Lung:152	2008.1–2010.3	152 (80, 72)	M = 94, F = 58	T: range: 60–77, median: 67.9 C: range: 60–76, median: 67.7
64	(Wei 2016) [97]	Shenmai Injection	Ginseng Radix, Ophiopogonis Radix	Injection	Lung:90	2014.5–2016.5	90 (45, 45)	M = 49, F = 41	T: 63.5 ± 5.3 C: 63.6 ± 5.3
65	(Wu et al. 2014) [98]	Shenmai Injection	Ginseng Radix, Ophiopogonis Radix	Injection	Lung:116	2011.2–2013.12	116 (57, 59)	M = 64, F = 52	T: 56.9 ± 8.5 C: 58.6 ± 7.8
66	(Dai et al. 2013) [99]	Kushen Injection	Sophorae Flavescentis Radix, Poria	Injection	Lung:106	2010.1–2012.12	106 (52, 54)	M = 59, F = 47	T: range: 38–72, mean: 58.13 C: range: 39–71, mean: 57.65
67	(Huang et al. 2013) [100]	Shenmai Injection	Ginseng Radix, Ophiopogonis Radix	Injection	Lung:26; breast:13; esophageal:5; colorectum:7; nasopharyngeal carcinoma:9	2008.6–2011.6	60 (30, 30)	M = 42, F = 18	T: 47.4 ± 3.6 C: 46.6 ± 4.3
68	(Wu 2014) [101]	Shenmai Injection	Ginseng Radix, Ophiopogonis Radix	Injection	Lung:21; breast:16; stomach:14; liver:19; esophageal:5; colorectum:40; nasopharyngeal carcinoma:5	2011.1–2013.9	120 (60, 60)	M = 68, F = 52	T: 58.6 ± 7.98 C: 58.1 ± 8.05
69	(Liang et al. 2012) [102]	Shenmai Injection	Ginseng Radix, Ophiopogonis Radix	Injection	Lung:36; breast:19; stomach:19; liver:26; colorectum:44; others:12	2010.2–2011.11	156 (86, 70)	M = 75, F = 81	T: 57.5 ± 8.57 C: 58.1 ± 9.05
70	(Wei 2021) [103]	Kanglai Te Injection	Coicis Semen	Injection	Others:86	2019.3–2021.6	86 (43, 43)	M = 53, F = 33	T: 51.08 ± 12.34 C: 52.37 ± 11.37
71	(Cui et al. 2022) [104]	Huangqi Sijunzi Decoction	Astragali Radix, Codonopsis Radix, Poria, Atractylodis Macrocephalae Rhizoma, Glycyrrhizae Radix	Decoction	Breast:94	2018.11–2020.12	94 (47, 47)	N/A	T: 45.20 ± 9.61 C: 47.83 ± 8.53
72	(Guglielmo et al. 2020) [32]	American ginseng	Panax Quinquefolius	Tablets	Head and neck:32	2018–2019	32 (17, 15)	M = 22, F = 10	T: range: 34–73, median: 58 C: range: 35–79, median: 55
73	(Jeong et al. 2010) [28]	Bojungikki-Tang	Astragali radix, Atractylodis lanceae rhizoma, Ginseng Radix, Angelicae Radix, Bupleuri Radix, Zizyphi fructus, Aurantii nobilis pericar-pium, Glycyrrhizae Radix, Cimicifugae Rhizoma, and Zingiberis Rhizoma	Granules	Lung:5; breast:11; stomach:5; colon:5; others:14	2009.5–2009.10	40 (20, 20)	M = 15, F = 25	T: 49.4 ± 10.8 C: 53.4 ± 8.0
74	(Gu et al. 2010) [105]	Shenfu Injection	Ginseng Radix, Aconitum carmichaeli Debx	Injection	Lung:11; breast:21; stomach:28; liver:12; colorectum:38; pancreatic:3	2005.9–2009.6	113 (63, 50)	M = 62, F = 51	T: 59.17 ± 8.5 C: 60.20 ± 9.66
75	(Oliveira et al. 2011) [29]	Guarana (Paullinia cupana)	Paullinia Cupana	Capsules	Breast:75	2008.2–2009.9	75 (43, 32)	M = 0, F = 75	T: 50.2 ± 11.95 C: 51.76 ± 9.73
76	(Kim et al. 2020) [33]	Korean red ginseng (KRG)	Panax ginseng C.A. Meyer	Pills	Colorectum:409	2013.12–2016.4	409 (206, 203)	M = 247, F = 162	T: range: 29–84; median: 60 C: range: 27–86; median: 60
77	(Barton et al. 2010) [27]	American ginseng	Panax Quinquefolius	Capsules	Lung:35; breast:109; colon:29; others:109	2005.10.20–2006.7.5	*282 (70, 72, 71, 69)	M = 96, F = 186	T1: 58 ± 11 T2: 60 ± 12 T3: 62 ± 11 C: 62 ± 13
78	(Sette et al. 2018) a [25]	PC-18 (Paullinia Cupana)	Paullinia Cupana	Capsules	Breast:43	N/A	43 (32, 11)	N/A	T: mean: 48.9 C: mean: 55.7d
	(Sette et al. 2018) b [25]				Breast:72	N/A	*72 (23, 24, 25)	N/A	T1: mean: 49 T2: mean: 51 C: mean: 52
79	(Lee et al. 2021) [34]	Sipjeondaebo-tang	Astragali Radix, Panax ginseng radix, Atractylodes Rhizoma Alba, Poria sclerotium, Rehmanniae Radix, Angelicae Gigantis Radix, Paeonia Radix, Cnidii Rhizoma, Glycyrrhizae Radix et Rhizoma, and Cinnamomi Ramulus	Granules	Lung:3; breast:19; gastrointestinal:18; head and neck: 5; urogenital: 5	2018.5–2020.6	50 (25, 25)	M = 13, F = 37	T: 56.6 ± 11.6 C: 58.7 ± 12.5
80	(Barton et al. 2013) [31]	Wisconsin Ginseng	Panax Quinquefolius	Capsules	Breast:206; colon:37; prostate: 14; hematologic: 17; hematologic: 12; others: 55	2018.10–2011.7	341 (171, 170)	M = 75, F = 266	T: 55.3 ± 12.7 C: 55.9 ± 11.8
81	(Costa et al. 2009) [26]	Guarana (Paullinia cupana)	Paullinia Cupana	N/A	Breast:36	N/A	36 (17, 19)	N/A	T: mean: 59 C: mean: 57

1. *M = 64, F = 38: this study calculated the number of males and females with some mistakes

2. (*M = 46, F = 38); (*M = 43, F = 30); (*M = 62, F = 52): the actual number of males and females recruited in these trials were unknown, these records referred to participants who completed experiment

3. (*T: 41.7 ± 11.4, C: 40.2 ± 11.7); (*T: 60.5 ± 11.8, C: 59.9 ± 9.3); (*T: 54.85 ± 5.3, C: 55.14 ± 5.27): the actual participants’ age in these trials were unknown, these records referred to participants who completed experiment

4. (*lung:23, breast:21, liver:20, cervical: 5, rectal:15): the actual number of cancer classification in these trials were unknown, these records referred to participants who completed experiment

5. *182 (62, 62, 58): T1: low dose group (30 mg/d); T2: high dose group (60 mg/d); C: control group

6. *282 (70, 72,71, 69): T1: high dose group (750 mg/d); T2: medium dose group (1000 mg/d); T3: low dose group (2000 mg/d); C: control group

7. *72 (23, 24, 25): T1: low dose group (7.5 mg); T2: high dose group (12.5 mg); C: control group

### Participants

A total of 7547 participants with CRF were recruited in the RCTs included in this review. After 312 participants dropped out or withdrew before the intervention was initiated, the remaining participants in 82 trials were allocated into the test groups (n = 3801) and the control or comparison groups (n = 3434). Excluding eight trials which did not report the number of males and females [25, 26, 38, 43, 54, 67, 104], 3399 males and 3195 females* (the number of males and females had some errors in one study [62]) were eventually included. Out of 82 RCTs, only 53 trials specifically reported the mean age and standard deviation of the participants in test group and control group, 20 trials reported the mean, median or range of age in two groups, seven trials reported the overall age characteristics of participants, and two trials did not report any information of age.

The top three categories of cancer in 82 trials were lung (participants: n = 2671; trials: n = 46), bowel (participants: n = 1532; trials: n = 34) and breast (participants: n = 1329; trials: n = 34), followed by stomach, liver, ovarian, cervical, esophageal, kidney, ureteral, pancreatic, gallbladder, nasopharyngeal carcinoma, head and neck or other categories. Regarding places of recruitment, 73 trials recruited participants from hospitals, one from newspaper or hospital advertisements [28], one from community cancer centers [31], one from center (not specified) [33], and six trials in five studies did not mention the places of recruitment [25–27, 76, 88].

### Diagnosis and included criteria

Information about the diagnosis and inclusion criteria of the CRF participants in this review is provided in Additional file 2: Table S2. Twenty-eight out of 82 trials regarded The International Statistical Classification of Diseases and Related Health Problems 10th Revision (ICD-10) as a preferred reference for CRF diagnostic criteria. Nineteen trials selected the relevant standards of Chinese medicine diagnostic criteria according to the Chinese Medicine Clinical Research of New Drugs Guiding Principles [11], Chinese Medicine Diagnosis [106], Chinese Medicine Internal Science [107], Practical Traditional Chinese Medicine Tumor Manual [108] or others.

Other scales such as PFS, KPS, ECOG, BFI, QLQ-C30, CFS*, NGFRS, VAS, a 11-point fatigue scale and a question about fatigue level were used as the tool to determine hte inclusion criteria for CRF participants in 52 trials. In particular, the score of KPS was set between 40 to 80 in 34 trials included in this review. Seven trials used ECOG with the score less than two. Seventy-two trials used tumor diagnosis and 10 trials had no information regarding the tumor diagnosis. One trial mentioned the level of HGB > 80 g /L [58], one trial limited the subjects with appropriate liver function (AST, ALT ≤ 2.5 X ULN), kidney function (Cr ≤ 1.5 X ULN) and the level of Hb ≥ 9 g/dL [33]. Three trials mentioned fatigue for at least one month [27, 31, 34].

In terms of life expectancy, 31 trials did not provide any information whereas 51 trials discussed different life expectancy periods, with more than 30 days in one trial, more 1–3 months in 33 trials, more than 4 months in one trial, more than or equal to 6 months in 15 trials and more than 9 months in one trial.

### Intervention

All the ingredients from each TCM preparation investigated in the included studies are summarized in Table 3. The TCM preparations tested in the included studies might be given in the form of injections (n = 34), decoctions (n = 31), capsules (n = 6), granules (n = 3), pills (n = 2), tablets (n = 1), oral solutions (n = 2), powder (n = 1), and decoction and injections (n = 1). One TCM preparation included did not provide information on formulation. Compared to the injections, most of the decoctions had no quality standards of the TCM in included RCTs. The names of the five most commonly studied TCM were *Kangai Injection* (n = 16) (康艾注射液), *Buzhong Yiqi Decoction* (n = 9) (補中益氣湯), *Shenqi Fuzheng Injection* (n = 6) (參芪扶正注射液), *Guarana* (*Paullinia cupana*) (n = 4) and *Shenfu Injection* (n = 3) (參附注射液).

At least 100 Chinese medicinal herbs were involved in the included trials for treating CRF and the 20 most common herbs were *Astragali Radix(*黃芪*) (n* = *48), Ginseng Radix(*人參*) (n* = *34), Codonopsis Radix(*黨參*) (n* = *30), Atractylodis Macrocephalae Rhizoma(*白朮*) (n* = *30), Glycyrrhizae Radix(*甘草*) (n* = *25), Poria (*茯苓*) (n* = *21), Angelicae Sinensis Radix (*當歸*) (n* = *21), Sophorae Flavescentis Radix* (苦參*) (n* = *17), Citri Reticulatae Pericarpium (*陳皮*) (n* = *15), Bupleuri Radi (*柴胡*) (n* = *12), Cimicifugae Rhizoma (*升麻*) (n* = *11), Ophiopogonis Radix (*麥冬*) (n* = *8), Rehmanniae Radix (*地黃*) (n* = *8), Dioscoreae Rhizoma (*山藥*) (n* = *7), Coicis Semen (*薏苡仁*) (n* = *7), Lycii Fructus (*枸杞*) (n* = *7), Ziziphus Jujuba Mill (*大棗*) (n* = *6), Paeoniae Radix Alba (*白芍*) (n* = *6), Pinelliae Rhizoma (*半夏*) (n* = *6) and Amomi Fructus (*砂仁*) (n* = *5)*.

### Control and comparison

In test group, the primary interventions were designed for TCM (n = 80) and TCM plus western medicine (n = 2). Thirty-seven trials used other treatment, 15 trials considered chemotherapy and 17 trials provided chemotherapy combined with other treatment as behavioral interventions. The primary interventions in control groups included placebo (n = 10), TCM (n = 2), western medicine (n = 3) and no treatment (n = 1). Thirty-nine trials used other treatment, 17 trials considered chemotherapy and 17 trials provided chemotherapy combined with other treatment as behavioral interventions. All the comparisons were described in Table 4 below.

**Table 4:** Intervention in test group and control group

Test group		Control group		Number of trials	Type of intervention
Primary intervention	Behavioral intervention	Primary intervention	Behavioral intervention		
TCM	CH	N/A	CH	14 trials	A1
		Placebo	CH	1 trial	A2
	CH + OT	N/A	CH + OT	17 trials	A3
	OT	TCM	OT	2 trials	A4
		Placebo	OT	2 trials	A5
		N/A	OT	32 trials	A6
	N/A	Placebo	N/A	7 trials	A7
		No treatment	N/A	1 trial	A8
		WM	CH	1 trial	A9
		N/A	CH	1 trial	A10
		N/A	OT	2 trials	A11
TCM + WM	OT	WM	OT	1 trial	B1
	N/A	WM	N/A	1 trial	B2

*TCM* traditional Chinese medicine; *WM* western medicine; *CH* chemotherapy; *OT* other treatment

A1: TCM + CH compare with CH; A2: TCM + CH compare with placebo + CH; A3: TCM + CH + OT compare with CH + OT; A4: TCM + OT compare with TCM + OT; A5: TCM + OT compare with placebo + OT; A6: TCM + OT compare with OT; A7: TCM compare with placebo; A8: TCM compare with no treatment; A9: TCM compare with WM + CH; A10: TCM compare with CH; A11: TCM compare with OT; B1: TCM + WM + OT compare with WM + OT; B2: TCM + WM compare with WM

### The intervention durations

The duration of the intervention ranged from 10 days to 16 weeks. The intervention durations were shorter than or equal to 1 month in 59 trials, between 1–2 months in 12 trials, between 2–3 months in 7 trials, more than 3 months in one trial, two cycles of treatment in 1 trial and four cycles of treatment in 1 trial. One trial did not provide any information regarding intervention duration.

### Outcomes

As shown in Table 5, the reported outcomes of the 82 trials included the primary outcomes (PFS and the scales of QoL) and the secondary outcomes (TCM syndrome score, Fatigue scales, other scales, cytokines test, blood test and others).

**Table 5:** Major findings of the RCTs included in the review

No	(Author, year)	Intervention	Duration	Outcomes		Efficacy				Safety	
				Primary outcome	Secondary outcome	Overall efficacy	Efficacy rate in test group	Efficacy rate in control group	Difference between the two groups	Non-serious	Serious
1	(Gu et al. 2021) [35]	A6	4 w	②	③④⑦	**√**	N/A	N/A	N/A	N/A	N/A
2	(Li et al. 2020) [36]	A6	14 d	①	③	**√**	67.70%	58.62%	P < 0.05	N/A	N/A
3	(Lin et al. 2020) [37]	A6	14 d	①	③⑥	**√**	91.00%	55.00%	P < 0.01	N/A	N/A
4	(Hu et al. 2020) [38]	A6	4w	②	④	**√**	N/A	N/A	N/A	AD1	AD1
5	(Wang et al. 2016) [39]	A6	28d	N/A	④⑥⑦	**√**	81.60%	48.60%	P < 0.05	N/A	N/A
6	(Zhao et al. 2011) [30]	A8	4w	②	⑤⑥⑧	**√**	N/A	N/A	N/A	AD2	No
7	(Cao 2020) [40]	A10	14d	①②	③⑥	**√**	96.67%	80.00%	P < 0.05	N/A	N/A
8	(Ou et al. 2022) [41]	A6	3 m	①②	⑤⑥⑦	**√**	N/A	N/A	N/A	AD3	No
9	(Zhang et al. 2018) [42]	A11	20d	N/A	④	**√**	92.30%	70.40%	P < 0.05	No	No
10	(Zhu et al. 2016) [43]	A1	28d	①②	④⑤⑧	**√**	N/A	N/A	N/A	N/A	N/A
11	(Wang et al. 2015) [44]	A1	2w	N/A	④⑦⑧	**√**	N/A	N/A	N/A	AD4	AD4
12	(Chen 2011) [45]	A6	20d	②	④	**√**	N/A	N/A	N/A	N/A	N/A
13	(Yang et al. 2015) [46]	A1	21d	①②	③	**√**	79.07%	44.19%	P < 0.05	AD5	No
14	(Kong et al. 2016) [47]	A1	42d	N/A	④⑦⑧	**√**	N/A	N/A	N/A	AD6	AD6
15	(Su et al. 2022) [48]	A6	6w	①②	③⑤	**√**	92.86%	46.43%	P < 0.05	N/A	N/A
16	(Jiang 2022) [49]	A1	84d	①②	⑥⑧	**√**	66.67%	40.00%	P < 0.05	N/A	N/A
17	(Luo et al. 2021) [50]	A6	8w	①②	N/A	**√**	N/A	N/A	N/A	N/A	N/A
18	(Wang et al. 2021) [51]	A6	10d	②	③④⑥	**√**	N/A	N/A	N/A	AD7	No
19	(He et al. 2020) [52]	A6	14d	①②	N/A	**√**	N/A	N/A	N/A	No	No
20	(Luo et al. 2019) [53]	A6	2w	①②	③	**√**	85.05%	68.60%	P < 0.05	AD8	AD8
21	(Liu 2018) [54]	A6	21d	①②	N/A	**√**	N/A	N/A	N/A	N/A	N/A
22	(Lin et al. 2018) [55]	A6	14d	①②	③	**√**	90.63%	53.13%	P < 0.05	N/A	N/A
23	(Li 2016) [56]	B2	14d	①	③⑧	**√**	70.00%	43.33%	P < 0.05	AD9	No
24	(Song et al. 2016) [57]	A3	14d	①②	N/A	**√**	N/A	N/A	N/A	N/A	N/A
25	(Liu et al. 2016) [58]	A3	21d	N/A	④	**√**	N/A	N/A	N/A	AD10	AD10
26	(Li 2016) [59]	A4	63d	①②	③⑥	**√**	76.71%	39.19%	P < 0.01	N/A	No
27	(Zhang et al. 2016) [60]	A6	28d	①②	③	**√**	92.31%	52.69%	P < 0.05	N/A	N/A
28	(Liang et al. 2016) [61]	A3	2 cycles	②	④	**√**	N/A	N/A	N/A	AD11	AD11
29	(Sun 2015) [62]	A1	4 cycles	①	⑧	**√**	58.30%	37.20%	P < 0.05	N/A	N/A
30	(Li et al. 2011) [63]	A1	12w	①	N/A	**√**	N/A	N/A	N/A	AD12	No
31	(Huang 2001) [64]	A6	20d	②	⑧	**√**	N/A	N/A	N/A	N/A	N/A
32	(Cao et al. 2022) [65]	A6	N/A	②	③④⑥	**√**	97.06%	76.47%	P < 0.05	N/A	N/A
33	(Zhang et al. 2019) [66]	A6	30d	N/A	③④	**√**	80.00%	46.67%	P < 0.05	N/A	N/A
34	(Wang 2019) [67]	A6	3w	②	④	**√**	N/A	N/A	N/A	N/A	N/A
35	(Yao 2019) [68]	A9	21d	②	③④	**√**	94.40%	77.10%	P < 0.05	N/A	N/A
36	(Chen et al. 2019) [69]	A3	4w	①②	N/A	**√**	N/A	N/A	N/A	N/A	N/A
37	(Wang 2018) [70]	A3	42d	①②	N/A	**√**	N/A	N/A	N/A	N/A	N/A
38	(Guo et al. 2017) [71]	A6	6w	①②	③⑦⑧	**√**	83.72%	56.82%	P < 0.05	N/A	N/A
39	(Gu et al. 2009) [72]	A3	20d	①②	N/A	**√**	N/A	N/A	N/A	N/A	N/A
40	(Liu et al. 2014) [73]	A6	30d	①	③	**√**	100.00%	48.00%	P < 0.01	N/A	N/A
41	(Li et al. 2013) [74]	A1	28d	②	③④⑦	**√**	78.38%	41.67%	P < 0.05	No	No
42	(Wang et al. 2016) [75]	A6	28d	N/A	④⑥⑦	**√**	76.60%	40.00%	P < 0.05	N/A	N/A
43	(Cai 2016) [76]	A6	30d	N/A	④	**√**	90.90%	76.40%	P < 0.05	N/A	N/A
44	(Ning et al. 2020) [77]	A4	12w	①②	⑦⑧	**√**	N/A	N/A	N/A	AD13	No
45	(Yang 2020) [78]	A11	30d	N/A	④	**√**	N/A	N/A	N/A	N/A	N/A
46	(Shan et al. 2020) [79]	A6	14d	①②	N/A	**√**	N/A	N/A	N/A	N/A	N/A
47	(Wu 2018) [80]	A6	1 m	N/A	④⑤	**√**	77.77%	48.13%	P < 0.05	N/A	N/A
48	(Shi 2017) [81]	A1	2w	①②	N/A	**√**	N/A	N/A	N/A	N/A	N/A
49	(Zhang et al. 2017) [82]	A6	30d	①	⑤	**√**	N/A	N/A	N/A	AD14	AD14
50	(Wang et al. 2015) [83]	A3	14d	②	③④	**√**	85.71%	54.29%	P < 0.01	N/A	N/A
51	(Lu 2014) [84]	A6	30d	①	N/A	**√**	64.70%	30.80%	P < 0.01	N/A	N/A
52	(Zhang et al. 2019) [85]	A6	21d	①②	③	**√**	88.24%	67.65%	P < 0.05	N/A	N/A
53	(Zhang et al. 2017) [86]	A3	28d	①②	③⑤	**√**	94.74%	68.42%	P < 0.05	AD15	AD15
54	(Wu et al. 2014) [87]	A6	4w	①②	N/A	**√**	N/A	N/A	N/A	N/A	N/A
55	(Feng 2014) [88]	A1	21d	①②	⑧	**√**	51.56%	34.38%	P < 0.05	AD16	AD16
56	(Zhang et al. 2012) [89]	A3	14d	②	④	**√**	N/A	N/A	N/A	AD17	AD17
57	(Li 2015) [90]	A1	42d	①②	③	**√**	N/A	N/A	N/A	N/A	N/A
58	(Yang et al. 2018) [91]	B1	4w	①②	③	**√**	N/A	N/A	N/A	N/A	N/A
59	(Ou et al. 2016) [92]	A1	2w	①②	N/A	**√**	N/A	N/A	N/A	AD18	AD18
60	(Zhao 2015) [93]	A1	2w	①	⑧	**√**	60.00%	42.50%	P < 0.05	N/A	N/A
61	(Leng 2015) [94]	A3	2w	①	⑧	**√**	40.00%	23.33%	P < 0.05	N/A	N/A
62	(Jing et al. 2010) [95]	A3	2w	②	N/A	**√**	15.90%	6.98%	P < 0.05	N/A	N/A
63	(Huang 2012) [96]	A3	14d	①②	⑧	**√**	52.50%	48.60%	P > 0.05	AD19	AD20
64	(Wei 2016) [97]	A6	4w	①	N/A	**√**	N/A	N/A	N/A	AD21	AD21
65	(Wu et al. 2014) [98]	A3	8w	①	③	**√**	N/A	N/A	N/A	N/A	N/A
66	(Dai et al. 2013) [99]	A3	2w	②	④	**√**	44.20%	16.70%	P < 0.05	AD22	AD22
67	(Huang et al. 2013) [100]	A3	42d	①②	③⑧	**√**	83.30%	63.30%	P < 0.05	AD23	N/A
68	(Wu 2014) [101]	A3	8w	①	③⑥	**√**	N/A	N/A	N/A	N/A	N/A
69	(Liang et al. 2012) [102]	A6	4w	①②	③⑥	**√**	N/A	N/A	N/A	N/A	N/A
70	(Wei 2021) [103]	A1	12w	N/A	④⑤	**√**	N/A	N/A	N/A	N/A	N/A
71	(Cui et al. 2022) [104]	A3	20d	①	N/A	**√**	N/A	N/A	N/A	AD24	AD24
72	(Guglielmo et al. 2020) [32]	A5	8w	N/A	④	** × **	N/A	N/A	N/A	N/A	N/A
73	(Jeong et al. 2010) [28]	A8	2w	②	④	**√**	N/A	N/A	N/A	AD25	No
74	(Gu et al. 2010) [105]	A6	3w	①	⑥⑦⑧	**√**	N/A	N/A	N/A	N/A	N/A
75	(Oliveira et al. 2011) [29]	A7	49d	②	④⑤	**√**	N/A	N/A	N/A	AD26	No
76	(Kim et al. 2020) [33]	A2	16w	②	④⑤⑥⑦	**√**	N/A	N/A	N/A	AD27	AD28
77	(Barton et al. 2010) [27]	A7	8w	②	④⑤	**√**	N/A	N/A	N/A	AD29	AD30
78	(Sette et al. 2018) a [25]	A7	21d	N/A	④	** × **	N/A	N/A	N/A	AD31	AD32
	(Sette et al. 2018) b [25]	A7	21d	N/A	④	** × **	N/A	N/A	N/A	AD33	No
79	(Lee et al. 2021) [34]	A5	3w	②	④⑤⑥	**√**	N/A	N/A	N/A	AD34	No
80	(Barton et al. 2013) [31]	A7	8w	N/A	④⑤	**√**	N/A	N/A	N/A	AD35	AD36
81	(Costa et al. 2009) [26]	A7	28d	N/A	④⑤	** × **	N/A	N/A	N/A	AD37	No

Intervention details were shown in Table 4 Type of intervention

①: PFS (Piper Fatigue Scale); ②: the scales of QoL (Quality of Life); ③: TCM syndrome score; ④: Fatigue scales; ⑤: Other scales; ⑥: Cytokines tests; ⑦: Blood tests; ⑧: Others

AD1: Test group: 19.44% (gastrointestinal adverse reactions: 5 cases, myelosuppression: 2 cases); Control group: 61.11% (gastrointestinal adverse reactions: 17 cases, myelosuppression: 5 cases)

AD2: dizziness: 4 case (16.0%); dry mouth: 3 cases (12.0%); diarrhea: 2 cases (8.6%); stomach discomfort: 2 cases (8.6%); nausea: 2 cases (8.6%); epistaxis: 1 case (4.0%); sore throat: 1 case (4.0%)

AD3: No obvious adverse reactions

AD4: Test group: myelosuppression: 70.97%; Control group: myelosuppression: 68.85%

AD5: Test group: leukopenia (I: 4 cases; II: 3 case); feel sick and vomit (I: 5 cases; II: 4 case); constipate (I: 4 cases; II: 0 case); Control group: leukopenia (I: 5 cases; II: 10 cases); feel sick and vomit (I: 8 cases; II: 12 case); constipate (I: 6 cases; II: 6 case)

AD6: Test group: myelosuppression: 70.00%; Control group: myelosuppression: 85.19%

AD7: No obvious adverse reactions

AD8: Test group: 6.54% (fever: 5 cases; drowsiness: 6 cases; stomatitis: 4 cases; rash: 3 cases), Control group: 20.93% (fever: 2 cases; drowsiness: 3 cases; stomatitis: 1 case; rash: 1 case)

AD9: No obvious adverse reactions

AD10: Test group: chemotherapy: feel sick and vomit: 30.6%; drop in hemoglobin: 16.8%; thrombocytopenia: 18.3%; leukopenia: 23.1%; none of the 41 patients in the treatment group had adverse reactions such as allergies during the treatment, and no obvious liver and kidney function damage was found. Control group: chemotherapy: feel sick and vomit: 65.3%; drop in hemoglobin: 19.6%; thrombocytopenia: 19.8%; leukopenia: 26.3%

AD11: Test group: the incidences of leukopenia, anemia, thrombocytopenia, feel sick and vomit were: 80.0%; 73.3%; 40.0%; 43.3%. Control group: the incidences of leukopenia, anemia, thrombocytopenia, feel sick and vomit were: 90.0%; 83.3%; 53.3%; 80.0%

AD12: Occasional vascular irritation or allergic reaction, no special adverse reactions occurred due to the use of TCM

AD13: No obvious adverse reactions

AD14: Test group: feel sick and vomit: 25.0%; rash, itching: 22.9%; myelosuppression: 18.8%; mucosal injury: 39.6%; Control group: feel sick and vomit: 39.6%; rash, itching: 41.7%; myelosuppression: 37.5%; mucosal injury: 58.3%

AD15: Test group: white blood cell drop: 47.37%; thrombocytopenia: 10.53%; decreased hemoglobin: 47.37%; diarrhea: 8.77%; feel sick and vomit: 29.82%; Control group: white blood cell drop: 49.12%; thrombocytopenia: 12.28%; decreased hemoglobin: 47.37%; diarrhea: 26.32%; feel sick and vomit: 45.61%

AD16: Test group: myelosuppression: 9.68%; gastrointestinal reaction: 22.58%; cardiovascular side effects: 6.45%; weak: 12.90%; abnormal liver function: 9.68%; skin reaction: 16.13%; Control group: myelosuppression: 28.13%; gastrointestinal reaction: 34.38%; cardiovascular side effects: 25.00%; weak: 43.75%; abnormal liver function: 21.88%; skin reaction: 18.75%

AD17: Test group: white blood cell drop: 38%; feel sick and vomit: 19%; neurotoxicity: 13%; hair loss: 13%; constipate: 0%; Control group: white blood cell drop: 67%; feel sick and vomit: 31%; Neurotoxicity: 33%; hair loss: 50%; constipate: 31%

AD18: Test group: myelosuppression: 17 cases; digestive tract reaction: 19 cases; fever: 10 cases; muscle ache: 20 cases; allergic reaction: 7 cases; neurotoxicity: 13 cases; Control group: myelosuppression: 7 cases; digestive tract reaction: 13 cases; fever: 6 cases; muscle ache: 15 cases; allergic reaction: 10 cases; neurotoxicity: 10 cases

AD19: Test group: white blood cell drop I II: 49 cases; thrombocytopenia I II: 16 cases; drop in hemoglobin I II: 14 cases; feel sick and vomit I II: 41 cases; peripheral neurotoxicity I II: 12 cases; phlebitis I II: 5 cases; abnormal liver function I II: 4 cases; kidney function I II: 1 case; abnormal ECG I II: 2 cases; hair loss I II: 72 cases; fever I II: 1 case; Control group: white blood cell drop I II: 37 cases; thrombocytopenia I II: 15 cases; drop in hemoglobin I II: 16 cases; feel sick and vomit I II: 39 cases; peripheral neurotoxicity I II: 14 cases; phlebitis I II: 7 cases; abnormal liver function I II: 9 cases; kidney function I II: 5 case; abnormal ECG I II: 4 cases; hair loss I II: 58 cases; fever I II: 1 case

AD20: Test group: white blood cell drop III IV: 19 cases (23.8%); drop in hemoglobin III IV: 1 case (1.3%); feel sick and vomit III IV: 7 cases (8.8%); hair loss III IV: 6 cases (7.5%); Control group: white blood cell drop III IV: 24 cases (33.3%); drop in hemoglobin III IV: 2 case (2.7%); feel sick and vomit III IV: 9 cases (12.5%); hair loss III IV: 6 cases (8.3%)

AD21: Test group: 35.56% (feel sick and vomit: 7 cases, leukopenia 9 cases); Control group: 60.00% (feel sick and vomit: 10 cases, leukopenia 12 cases, Peripheral nerve paresthesia 5 cases)

AD22: Test group: myelosuppression: 13 cases; digestive tract reaction: 16 cases; fever: 5 cases; muscle ache: 21 cases; allergic reaction: 9 cases; neurotoxicity: 12 cases; Control group: myelosuppression: 21 cases; digestive tract reaction: 27 cases; fever: 16 cases; muscle ache: 28 cases; allergic reaction: 14 cases; neurotoxicity: 18 cases

AD23: Test group: leukopenia I: 2 cases, II: 4 cases; feel sick and vomit I: 4 cases, II: 6 cases; Control group: leukopenia I: 6 cases, II:12 cases; feel sick and vomit I: 9 cases, II: 11 cases; peripheral nerve paresthesia I: 4 cases

AD24: Test group: 4.25% (2/47); Control group: 6.38% (3/47)

AD25: Two patients reported minor adverse effects including increased flatulence and dyspepsia and these were assessed as grade 1 on the NCI-CTC scale

AD26: All reported adverse effects were Common Terminology Criteria for Adverse Events v3.0 Grade 1 only. Test group: insomnia: 22 cases palpitation: 10 cases; nausea: 25 cases; anxiety: 17 cases; dermatologic: 1 case; Control group: insomnia: 31 cases; palpitation: 12 cases; nausea: 35 cases; anxiety: 25 cases; dermatologic: 3 cases

AD27: Adverse events of any grade were observed in a total of 366 patients (KRG, 86%; placebo, 86%)

AD28: Severe adverse events (≥ grade 3) occurred rarely and equally in both groups, except neutropenia. Test group: nausea: 11 cases; decreased appetite: 1 case; neutropenia: 28 cases; diarrhea: 5 cases; thrombocytopenia: 2 cases; peripheral neuropathy: 1 case; leukopenia: 2 cases; stomatitis: 1 case; headache: 1 case; vomiting: 2 cases; Control group: nausea: 11 cases; decreased appetite: 3 cases; neutropenia: 15 cases; diarrhea: 1 case; leukopenia: 1 case; stomatitis: 1 case

AD29: agitation I II: 26 cases; anxiety I II: 40 cases; insomnia I II: 103 cases; nausea I II: 83 cases; vomiting I II: 34 cases

AD30: agitation III: 1 case; anxiety III: 1 case; insomnia III: 8 cases; nausea III: 5 cases; vomiting III: 6 cases

AD31: Test group: anxiety I II: 1 case; epigastric pain I II: 2 cases; tachycardia I II: 1 case; Control group: insomnia I II: 3 cases; anxiety I II: 1 case; epigastric pain I II: 2 cases; tachycardia I II: 1 case

AD32: Test group: insomnia III IV: 1 case; Control group: insomnia III IV: 3 cases

AD33: Test group: mucositis I II: 1 case; Epigastric pain I II: 1 case; Control group: insomnia I II: 2 cases

AD34: Test group: dyspepsia I: 1 case; Control group: pruritus I: 1 case

AD35: Test group: nausea: 5 cases; vomiting: 2 cases; insomnia: 9 cases; anxiety: 4 cases; agitation: 2 cases; Control group: nausea: 3 cases; vomiting: 2 cases; insomnia: 10 cases; anxiety: 5 cases; agitation: 4 cases

AD36: Insomnia III: one case each in the test group and the control group

AD37: No significant toxic effects from test group

#### Primary outcome

- PFS was considered a measuring tool for CRF and was selected by more than half of the trials (n = 46).
- In addition, scales of QoL were selected in 51 trials, and 9 trials employed more than two scales. The most common scales included KPS (n = 27) and QLQ-C30 (n = 17), other were PSQI (n = 3), SF-36 (n = 3), FACIT-F (n = 3), GQoLI-74 (n = 2), FACT-F (n = 2), FACT-G (n = 1), FACT-ES (n = 1), Quality of Life Questionnaire (n = 2), but 31 trials did not provide any information.

#### Secondary outcome

- Twenty-seven trials selected TCM syndrome score.
- Fatigue scales included BFI or BFI-C (n = 17), CFS (n = 5), BFI combine CFS (n = 1), BFI combine MFSI-SF (n = 1), BFI combine CFS* (n = 4), FSI (n = 3), FSI combine MFSI-SF (n = 1), NCCN GFRS (n = 1), VAS-F combine TOI-F (n = 1).
- Other less commonly used scales were HADS (n = 5), GIC combine LASA (n = 1) and HADS combine SDS (n = 1). The scales of BDI, CS, GCSG, LCSG, POMS, PSS, SS were only used once.
- For the cytokines test, 15 trials reported the results of CD3^+^, CD4^+^, CD8^+^, CD3^+^CD4^+^, CD3^+^CD8^+^, CD4^+^/CD8^+^, CD16^+^56^+^, TNF-α, IFN-γ, IL-1, IL-1β, IL-2, IL-4, IL-5, IL-6, IL-8, IL-10, NK cell, IgG, IgA, IgM and TGF-β.
- Out of the 11 trials that reported the results of blood test, 5 trials conducted HGB test, WBC combined PLT were reported in three trials, COR and DM were separately reported in two trials, and the remaining parameters including Alb, TP, RBC, MCH, MCHC, MCV, HCT were only reported once. Seventy-one trials did not provide any information regarding blood test results.
- Other outcomes included RECIST (n = 5), weight (n = 2), cardiac function (n = 2), liver function (n = 2), kidney function (n = 2), rhG-CSF dosage (n = 2), blood viscosity (n = 1), urinalysis (n = 1), physical and chemical indicators (n = 1), improvement in cancer cachexia-related symptoms (n = 1), standards for the diagnosis and treatment of common malignant tumors in China (n = 1).

### Efficacy

The results of the included trials showed that 78 trials reported an overall efficacy on the use of TCM in treating CRF whereas only 4 trials showed no effect.

Out of the 34 trials which demonstrated the efficacy rate in test groups as compared to control groups, 28 trials showed statistically significant difference (p < 0.05), 5 trials showed statistical differences at a two-sided P-value less than 0.01, while one trial showed the negative difference (p > 0.05). In particular, the efficacy rate in test groups ranged from 15.9 to 100%, 11 trials were more than 90%, 20 trials were over 50% and less than 90% and 3 trials were below 50%. The efficacy rate in control groups ranged from 6.96 to 80% with 15 trials over 90% and 19 trials below 50%. Based on the different classification of TCM dosage forms, the efficacy rate of test groups considering TCM injections ranged from 15.9 to 92.31% while for TCM decoctions in 15 trials, the range was 70.00 to 100.00%.

The three most used TCM products, such as *Kangai Injection* (n = 16), *Buzhong Yiqi Decoction* (n = 9) and *Shenqi Fuzheng Injection* (n = 6), all show overall efficacy in this review. In 16 trials, *Kangai Injection* not only effectively improved the scores of PFS, BFI, FSI, QLQ, HAMA, SDS and other scales, but also improved the level of IL-1β [49, 51, 62, 78–84, 89, 92–96]. The interventions in the test group included TCM plus OT (5 trials) [51, 79, 80, 82, 84], TCM plus OT plus CH (5 trials) [83, 89, 94–96], TCM plus CH (5 trials) [49, 62, 81, 92, 93] and only TCM (1 trial) [78]. A total of nine studies reported significant differences in the test group and the control group. The efficacy rate of the test group ranged from 15.90 to 85.71%, while the efficacy rate of the control group was lower than 55%.

Among 9 included RCTs, *Buzhong Yiqi Decoction* was not only used in China [38, 43, 45, 55, 58, 67, 76, 91], but also widely considered as *Bojungikki-Tang* in traditional Korean medicine [28], and the results all showed that this decoction had a good curative effect in the treatment of CRF. Two studies reported the efficacy rate of between the control group and the test group, with the efficacy rate as high as 90% in the test group [55, 76].

In 6 included trials studying *Shenqi Fuzheng Injection* [36, 53, 60, 70–72], PFS was used to measure the change of fatigue level and the results of one trial showed that physical fatigue might be improved, while other findings suggested that the four fatigue levels of PFS were improved in the test group. Four studies reported the efficacy rates after treatment with this injection, ranging from 67.7 to 92.31% in the test group and 52.9 to 68.6% in the control group [36, 53, 60, 71].

One trial found that *Zini Yiqiyangxue Recipe* (自擬益氣養血方) was fully effective in treating patients with colorectal cancer fatigue, with the overall efficacy rate of 100% in the test group as compared to 48% in control group (p < 0.01). After the intervention, it was reported that 9 cases were obviously effective and 16 cases were effective in the test treatment group, and nearly half of the control group were ineffective (13/25) [73].

The efficacy of *American ginseng (Panax quinquefolius)* and *Guarana* (*Paullinia cupana*) should be carefully considered in this review. Two trials that considered *American ginseng* as a TCM intervention had mixed results in terms of overall efficacy [27, 32]. One trial with *American ginseng* alone improved fatigue and QoL [27], but the other trial combining *American ginseng* with other treatments did not significantly improve BFI scores [32]. For *Guarana*, only 1 of 4 trials in this review supported its use for fatigue relief [29], and the other three trials did not show an overall efficacy of the treatment [25, 26]. In particular, a study reported two randomized, double-blind trials involving standardized dry-purified extract of *Paullinia cupana* (PC-18). One trial compared PC-18 (37.5 mg orally twice daily) with placebo, while another trial compared either 7.5 mg or 12.5 mg of PC-18 with placebo, and the results of both trials showed no statistical difference between the two groups [25]. Similarly, a clinical trial of 36 breast cancer patients receiving adjuvant radiation therapy did not show a significant difference between the *Guarana* group (75 mg orally per day) and placebo with the scores of CFS*, BFI or BDI [26]. However, one trial showed that *Guarana* (50 mg orally twice daily) significantly improved FACIT-F, FACT-ES, and BFI scores compared to placebo on day 21 and day 49 (p < 0.01) [29].

### Safety

Among the 82 trials included, only 3 trials reported no adverse reactions [42, 52, 74], Forty-six trials did not provide any information about adverse reactions. One trial reported no serious adverse effects but did not reported any general adverse reactions [59]. For the other 32 trials (39.02%) that reported the adverse effects, 5 trials showed no obvious adverse reactions [26, 41, 51, 56, 77], and 27 trials reported specific symptoms with the number of cases. Non-serious adverse effects mainly included gastrointestinal discomfort (such as diarrhea, nausea, vomiting or constipate), myelosuppression (leukopenia, thrombocytopenia, or erythrocytopenia) and other mild complaints including anxiety, insomnia, fever, dizziness, rash, etc. The various forms of TCM preparations tested also drew further attention to the safety considering that, among the included trials that reported adverse reactions, 12.2% (10/82) of the trials used TCM injections while 6.09% (5/82) used TCM decoctions.

Only 5 trials reported several serious adverse reactions (Common Terminology Criteria for Adverse Events level ≥ 3) during TCM treatment in the test group [25, 27, 31, 33, 96]. The most frequently encountered serious adverse symptoms were nausea (or vomiting) (n = 28 cases), neutropenia (n = 28 cases), leukopenia (n = 21 cases), hair loss (n = 8 cases) and insomnia (6 cases). Due to TCM and chemotherapy being considered interventions at the same time, a trial using Kangai Injection showed more serious side effects [96]. In four other trials, *Korean red ginsen*g (KRG) [33], *American ginseng* [27], *Paullinia cupana* [25] and *Wisconsin Ginseng* [31] were used alone with a few serious side effects. Overall, no clear conclusions can be made about the safety of TCM due to inadequate reporting on adverse reactions in trials included in this systematic review.

### CONSORT-CHM

The summary of the CONSORT-CHM quality assessment results of the 82 RCTs is shown in Additional file 2: Table S3. None of the trials fully met all the CONSORT-CHM criteria. The items of randomization, allocation, implementation, blinding, ancillary analysis, harms, limitations, registration, protocol, funding were mostly lacking relevant information. For the random sequences, 46 trials did not provide any information, 9 trials partially and 27 trials fully reported information about randomization, in which 21 trials used random number tables, 4 trials used computer-generated randomization, one trial considered central randomization, and one used the method of sealed envelope. Only 4 trials specifically introduced allocation in detail. Four trials referred to the design of clinical trial implementation, for whom generated the random assignment sequence, enrolled the participants, and assigned the participants to the intervention groups. Seventy-five trials had no information on blinding. Only seven trials mentioned the blinding methods, but lacked a detailed introduction. Six trials used ancillary analysis, including correlation analysis (n = 2), cross experiment (n = 3), and subgroup analysis (n = 1). For the harms, 36 trials reported some information while 47 trials did not. Among the 16 trials that reported limitations of RCTs, only 7 trials specifically mentioned disadvantages in designing experiments whereas the remaining 9 trials lacked a detailed description on limitations. There were only 3 articles reporting details of registration, protocol, and funding.

### Risk of bias

**Fig. 2:**
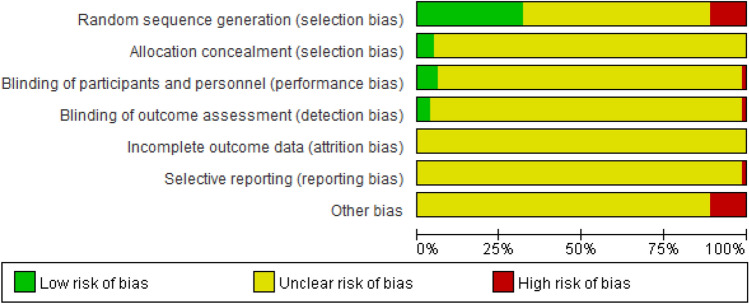
Risk of bias graph: the judgements of the review authors about each risk of bias item presented as percentages across all included studies

The six domains of the risks of bias evaluation for included trials are shown in the following and the summary of the risk of bias assessment is shown in Fig. 2. Detailed evaluation of the risk of bias of eac included study is provided in the Additional file 2: Fig. S1a, S1b.

#### Random sequence generation (selection bias)

- Twenty-six trials were at a low risk of bias due to the application of random sequence generation, 9 trials were deemed having a high risk of bias based on admission order, hospital number, medical record number or other order [37, 39, 40, 49, 67, 72, 75, 95, 103]. The remaining 47 trials had no specific information.

#### Allocation concealment (selection bias)

- Four trials reported detailed information were regarded as low risk of bias [27, 29, 33, 34], and 78 trials were regarded as unclear risk of bias.

#### Blinding of participants and personnel (performance bias)

- Five trials reported using double-blinded design were regarded as low risk [27, 29, 32–34] and one trials were at a high risk of bias for blinding patients only [37]. The remaining 76 trials did not mention the blinding of participants and personnel.

#### Blinding of outcome assessment (detection bias)

- Three articles mentioned blinding of outcome measurers [29, 33, 34]. One trial did not blind the outcome measurers and was therefore regarded as high risk of bias [37]. Seventy-eight trials had unclear information.

#### Incomplete outcome data (attrition bias)

- All trials did not provide detailed information.

#### Selective reporting (reporting bias)

- Since results of some scale points were not presented in a trial, it was regarded as having high risk of bias. The remaining 81 trials were evaluated at an unclear risk of bias.

#### Other bias

- There was no baseline population characteristics in a trial [54]. One trial had a problem with the statistics of the number of male and female [62], six trials did not mention the inclusion and exclusion criteria for participants [67, 70, 80, 85, 88, 92] and result statistics of one trial was wrong [100].

## Discussion

This systematic literature review included 82 RCTs which assessed the efficacy and safety of TCM for the treatment of CRF. Regarding efficacy, 78 studies reported an overall efficacy of TCM in treating CRF compared with the control group regardless of whether behavioral interventions were involved, in which 33 trials demonstrated that the efficacy rate was statistically significant (p < 0.05 or p < 0.01). Regarding the safety of TCM under investigation, non-serious or reversible adverse effects associated with the use of TCM for CRF had been reported. Among the included studies, TCM which demonstrated significant benefits on the management of CRF could be either herbs (including *Astragali Radix (*黃芪*), Ginseng Radix (*人參*)* and *Codonopsis Radix (*黨參*)*) or finished products (including *Kangai Injection* (康艾注射液), *Buzhong Yiqi Decoction* (補中益氣湯) and *Shenqi Fuzheng Injection* (參芪扶正注射液)). The benefits of TCM were reported in terms of improvements in the scores of the fatigue scales as well as physical indicators (e.g., cytokines level, blood tests). Overall, the findings of this review suggested that some TCM were effective in the management of CRF and relatively safe to use. However, the positive findings of the current review should be interpreted with cautions due to the concerns about the quality of the included RCTs. The implications of the review findings about TCM for CRF on clinical practice and the methodology of RCTs will be discussed in further details in the following.

### Efficacy of TCM herbs for CRF

Among the included studies in this review, more than 100 herbs, whether used as single herb alone or as a combination of herbs, were under investigation for their potential role in treating CRF. The three most common herbs which demonstrated benefits on CRF were *Astragali Radix (*黃芪*)* (n = 48), *Ginseng Radix (*人參*)* (n = 34) and *Codonopsis Radix (*黨參*)* (n = 30). From the perspective of TCM theory, these three herbs are the representative herbs of tonifying *qi* [109]. *Qi* is the driving force of biological activity in the human body and plays a key role in body metabolism and immunity, which may help explain the mechanism of TCM in treating fatigue [15]. For cancer patients, radiation therapy and chemotherapy are regarded as heat toxicity, which may lead to spleen-*qi* deficiency and accumulation of dampness. Therefore, the herbs which promote and nourish the balance between *yin* and *qi* are often recommended in the treatment for CRF [110].

In terms of the pharmacological mechanism, the two major pathological reactions of CRF are inflammatory immune and metabolic disorders [111]. Similarly, these 3 herbs have demonstrated certain immunological regulatory and anti-tumor activities and the effect of reducing the adverse toxicity of chemotherapy or radiotherapy in the clinical treatment [112–114]. For example, *Astragaloside IV,* as an active extract of *Astragali Radix*, could inhibit the growth and proliferation of tumor cells and induce apoptosis of tumor cells [115], and promote immunity by regulating levels of IL-1β, IL-6, and TNF-α cytokines [116]. *Ginsenosides*, the main active agents in *Panax ginseng,* could help boost immunity by altering the levels of various cytokines [117, 118]. It was also reported that *Ginseng Radix* was associated with reduced inflammatory processes and released of cortisol through the stress axis and therefore could benefit CRF [119]. *Codonopsis Radix* had the characteristics of *Gan* and *Ping*, tonifying the spleen and lung, nourishing blood, stimulating fluid, and strengthening body’s resistance [120]. As reported, it had a beneficial effect on improving the clinical symptoms and QoL of cancer patients [113]. Toxicological studies had been conducted on various extracts of *Codonopsis Radix*, and few significant toxicity and side effects had been observed or recorded [121].

### Efficacy of TCM products for CRF

In addition to some herbs, a variety of TCM products were investigated for their role in the treatment of CRF. Among the TCM products which showed efficacy, the most researched products were *Kangai Injection* (康艾注射液), followed by *Buzhong Yiqi Decoction* (補中益氣湯) and *Shenqi Fuzheng Injection* (參芪扶正注射液). These products composed of the three herbs mentioned above (*Astragali Radix*, *Ginseng Radix* and *Codonopsis Radix*), and their benefits could be partly explained with the functions of tonifying *qi* and supporting righteousness, enhancing the body's immunity and improving fatigue.

*Kangai Injection*, a famous TCM injection, mainly consists of *Astragali Radix, Ginseng Radix* and *Sophorae Flavescentis Radix*. Network pharmacological indicated that some of the active extracts, such as oxymatrine, ginsenoside, and kaempferol, could regulate the pharmacological activity of TP53, TNF, VEGFA, EGFR or other key targets, and also mediate cancer, TNF, HIF-1, PI3K-Akt and some signaling pathways [122, 123]. It was also reported that this product could enhance the body's immune system without causing damage to the body's normal cells [124]. The product could also improve the clinical effectiveness of lung, colorectal, breast or other cancer patients and was reportedly beneficial to the QoL and physical condition of the patients [115, 125, 126] without causing any significant adverse reactions [127].

*Buzhong Yiqi Decoction* (also known as *Hochuekki-to* or *TJ-41* in Japanese, or *Bojungikki-Tang* in Korean) mainly composes of eight herbs including *Astragali Radix, Atractylodis Macrocephalae Rhizoma, Ginseng Radix, Glycyrrhizae Radix, Angelicae Sinensis Radix, Bupleuri Radi, Citri Reticulatae Pericarpium* and *Cimicifugae Rhizoma*. As a traditional Chinese decoction, it mainly played the role of regulating the spleen and stomach and ascending *qi* and *yang* [112, 128, 129]. In recent years, some studies have shown that *Buzhong Yiqi Decoction* had strong immunomodulatory and anticancer effects [130, 131]. Based on clinical experiences, this decoction could also prevent immunosuppression caused by surgical stress, improve the QoL of patients with CRF, and reduce side effects caused by radiotherapy or chemotherapy such as leukopenia [113].

*Shenqi Fuzheng Injection* mainly composes of *Astragali Radix* and *Codonopsis Radix* [132] and has been approved by China's FDA since the 1990s. The main function of this product is to tonify *qi* and support righteousness mostly for symptoms caused by deficiency of the lung and spleen. It can also be considered as an adjuvant therapy of lung cancer, breast cancer and colorectal cancer [133–136]. Improvement in the QoL and immunity of cancer chemotherapy patients and reduced adverse reactions during the treatment of cancer associated with the use of this preparation had been previously reported [112, 133, 134, 137].

Similarly, this systematic review showed that these three TCM products might have a positive effect on CRF by improving some fatigue scale scores, cytokines level and blood tests compared with control group, and most patients showed no obvious adverse reaction after medication. Some findings also supported the anti-fatigue efficacy of these products in CRF patients [128, 138]. According to the findings of this review, *Kangai Injection, Buzhong Yiqi Decoction* and *Shenqi Fuzheng Injection* are the TCM prescriptions worth considered for their role and potential benefits in treating CRF.

### Safety of TCM for CRF

Apart from efficacy, the evaluation of the safety of TCM was another aspect of this review. Although the current evidence that some herbs improve the fatigue in cancer patients is strong, some herbs, even when used alone, should be considered with caution, such as *American ginseng (Panax quinquefolius)* and *Guarana (Paullinia cupana)*. Third-degree adverse reactions with *American ginseng* have been reported in rates ranging from 1 to 5% for agitation, insomnia, nausea, and vomiting [139]. For *Guarana*, depending on the different intervention doses and outcome measures, this herb might be effective or ineffective in alleviating fatigue symptoms [140]. Currently, the available evidence cannot accurately confirm the efficacy and safety of these two herbs as a TCM treatment for CRF. In addition, incomplete processing or long-term usage of large doses of TCM will also aggravate the harm [141]. For example, excessive use of certain herb for invigorating *qi* could damage the vitality of the body, produce dryness and heat, and cause symptoms of “*Shang huo*” in TCM theory [142]. The risk of drug-herb interactions should also be taken into consideration, and the combination of herbs and some prescription medicines (such as central stimulants and antipsychotics) should be used with caution or even avoided. [142–144].

However, compared to other TCM preparations, the risk of adverse effects associated with the use of injections was more concerning [145]. Adverse drug reactions (ADR) related to TCM injections accounted for more than 50% of the total ADR reported in the use of TCM [146]. Common adverse reactions included rash, itching, chills, fever, abdominal pain, etc. Allergic reactions and shock could also occur in some severe cases. Bacterial endotoxin (or pyrogen), abnormal toxicity, visible foreign matter, insoluble particles, residual solvents, and other substances were considered as safety indicators for detection. Besides, the composition of TCM injection is complex and diverse, such as multiple extracts or unknown chemicals from plant/animal sources, which increase the difficulty of scientific quality monitoring and control [146]. Continuous research effort to evaluate the efficacy and safety of TCM injections and to standardize the quality control system is prominent for promoting evidence-based clinical use of TCM injection in CRF.

### Improvement of RCTs design

Due to the insufficient evidence and high or unclear risk of bias of included RCTs, recommending TCM for CRF patients is a topic worthy of consideration. Further analysis of trials included in this review identified common limitations and provided relevant insights for designing and improving the quality of RCTs.

Firstly, the overall quality of RCTs studies included in this review was generally poor, a finding commonly reported in previous systematic reviews involving TCM for other conditions [147–150]. Among 82 included trials, none of them were completely in compliance with CONSORT-CHM guideline. Based on the Cochrane risk-of-bias tool, two major considerations for risk of bias were randomization generation and blinding. However, in this review, only 27 (32.93%) trials fully reported the mechanism of randomization generated via random sequence, other trials only addressed randomization using phrases including “randomization into two groups” or “in order of admission”. At the same time, only 7 (8.54%) trials mentioned blinding procedures in this review. Some research indicated that efficacy rate was overestimated by 17% in RCTs with no blinding as compared to those with blinding. Unclear or inadequate allocation concealment overstated the effect by 30–41% as compared to those with allocation fully hidden [151]. Therefore, it is meaningful and necessary to fully report the details of randomization and blinding by referring to the CONSORT-CHM statement and Cochrane guidelines.

Secondly, the limited sample size of included RCTs is also a concern. In this review, only 3 trials (3.66%) had more than 200 participants in the clinical studies [27, 33, 63] and 14 trials (17.07%) had a sample size between 100 and 200. Most of the trials had a limited number of subjects with sample size of less than 100 in 65 trials (79.27%), and three studies with less than 20 patients assigned to the test and control groups [26, 32, 89]. If the sample size is too small in clinical trials, it is difficult to observe related occurrence and progression of the disease outcomes. On the contrary, a sample size which is too large will lead to a waste of time, resource, and money [152, 153]. When designing the study, expected sample size should be indicated, either based on accurate calculations or practical limitations. According to the CONSORT statement, it is essential to calculate and report the sample size in all published RCTs. Sample size calculation does not only allow researchers to identify the endpoint, but also inform readers about some possible adaptation to actual practice, such as performing further analysis or advance trial termination [152]. Therefore, the quality and validity of studies will be greatly improved if all important details of sample size characteristics and calculations are considered and presented.

Thirdly, most of trials included in this review treated scale score changes as an outcome indicator to estimate the efficacy of using TCM. More than half of trials considered PFS (47/82) and QoL scales (51/82). Some other types of scales, including TCM syndrome score and fatigue scales, were also widely used. Currently, researchers aim to investigate more evidence for CRF, and a large number of fatigue scales are gradually appearing in the current research to assess psychological, physiological, endocrine, metabolic and other indicators of patients [154, 155]. But there is still no single authoritative evaluation tool and diagnostic method [156]. Due to the complex pathogenesis and multiple correlation factors of CRF, it is difficult for related scales to comprehensively assess the patient's symptoms, disease duration and intensity [157]. Therefore, in this review, repeatability and reliability of test results cannot be fully guaranteed in included trials with incomplete reporting. Based on the CONSORT statement and Cochrane guidelines, the scale items reported should be as specific as possible to eliminate any concerns about incomplete result data or selective reporting bias.

### Limitations

There were several limitations in this review. Firstly, we only focused on RCTs, and excluded other study design and intervention approaches, resulting in limited consideration of the evidence for the treatment of CRF. Secondly, this review analyzed data from primary sources and the quality of trials was found to be low upon evaluation. There was some risk of bias in trials investigating the evidence on the efficacy and safety of TCM for CRF. Thirdly, no accurate diagnostic criteria for CRF patients were considered as multiple and incomplete diagnostic criteria were reported in the included RCTs. Finally, in TCM, there is often more than one ingredient to exert the therapeutic effect. The mutual induction or inhibition of various ingredients will also have an impact on the final curative effect. In our study, however, we only examined a few representative herbs and lacked an in-depth exploration of the inner relationship among other ingredients, which could result in bias when suggesting the herbs and products. Therefore, further studies need to improve on the quality of evidence, to establish the network analysis between relevant herb-ingredient-pharmacological activity of TCM for CRF, and to emphasize more on the efficacy of TCM.

### Improvement for further research

Based on further analysis of RCTs included in this review, common limitations and improvement were identified. The following summarizes insights into developments in the field of TCM treatment of CRF. (1) In order to standardize the use of TCM in the diagnosis and treatment of CRF, unified comprehensive diagnosis and efficacy measurement standards should be established; (2) multicenter, large-sample RCTs of CRF should be performed to minimize bias caused by age and sex differences and to ensure the validity of the results; and (3) clinical studies of multicomponent therapies should be conducted to enrich the TCM studies of CRF and at the same time verify whether it has a comparative advantage over a single components in terms of safety and reliability.

## Conclusion

Based on this review, the included TCM approaches have shown to be beneficial in the management and treatment of CRF. However, no recommended conclusion on the efficacy and safety of TCM in the treatment of CRF patients could be drawn as there were some concerns over the quality and bias of relevant RCTs. It is fundamental to standardize the diagnosis and treatment of CRF, improve RCTs protocol and conduct more clinical studies to present convincing evidence and provide an updated reference for disease medication in the future.

### Supplementary Information

**Additional file 1.** A detailed description of each search strategy.

**Additional file 2: Table S1.** Level of risks of bias. **Table S2.** Diagnosis and included criteria of the CRF participants in RCTs in this review. **Table S3.** Evaluation of included trial studies using the CONSORT-CHM statement. **Figure S1a.** Risk of bias summary: review authors' judgements about each risk of bias item for each included study (Study No. 01-42). **Figure S1b.** Risk of bias summary: review authors' judgements about each risk of bias item for each included study (Study No. 43-81).

## Data Availability

All data are fully available without restriction.

## Abbreviations

CRF: Cancer-related fatigue
TCM: Traditional Chinese medicine
RCTs: Randomized controlled trials
CNKI: China National Knowledge Infrastructure
WOS: Web of science
PRISMA: Preferred reporting items for systematic reviews and meta analyses
CONSORT-CHM: Consolidated standards of reporting trials statement extensions for Chinese herbal medicine formulas
WM: Western medicine
CH: Chemotherapy
OT: Other treatment
CH + OT: Chemotherapy plus other treatment
MeSH: Medical subject headings
ICD-10: The International Statistical Classification of Diseases and Related Health Problems 10th Revision
KRG: Korean Red Ginseng
ADR: Adverse drug reactions
PFS: Piper fatigue scale
QoL: Quality of life
WHO: World Health Organization
KPS: Karnofsky performance status
QLQ-C30: EORTC quality of life questionnaires
GQoLI-74: Generic quality of life inventory-74
SF-36: 36-Item short-form
FACT-F: Functional assessment of cancer therapy–fatigue
FACT-G: Functional assessment of cancer therapy–general
FACIT-F: Functional assessment of chronic illness therapy–fatigue
FACT-ES: Functional assessment of chronic illness therapy–endocrine symptoms
PSQI: Pittsburgh sleep quality index
QoL^1^: Quality of life questionnaire
QoL^2^: Quality of Life Questionnaire and Quality of life score (QoL) for cancer patients (Chinese version draft)
BFI: Brief fatigue inventory
CFS: Cancer fatigue scale
CFS*: Chalder fatigue scale
MFSI-SF: Multidimensional fatigue symptom inventory–short form
FSI: Fatigue symptom inventory
BFI-C: Chinese version of brief fatigue inventory
TOI-F: Trial outcome index–fatigue
VAS-F: Visual analogue scale of global fatigue
NGFRS: NCCN guidelines fatigue rating scale
HADS: Hospital anxiety and depression scale
POMS: Profile of mood states
HAMA: Hamilton anxiety scale
SDS: Self-rating depression scale
SS: Satisfaction survey
CS: Comfort survey
PSS: Perceived stress scale
GIC: The global impression of change
LASA: Linear analogue self assessment scale
BDI: Beck inventory depression scale
GCSG: Gastric cancer symptom grading scale
LCSG: Lung cancer symptom grading scale
HGB: Hemoglobin
Alb: Albumin
WBC: White blood cell
TP: Total protein
PLT: Platelet
COR: Cortisol
RBC: Red blood cells
MCH: Mean corpuscular hemoglobin
MCHC: Mean corpuscular hemoglobin concentration
MCV: Mean corpuscular volume
HCT: Hematocrit
DM: Degree of myelosuppression
rhG-CSF: Recombinant human granulocyte colony stimulating factor
RECIST: Response evaluation criteria in solid tumors
AST: Enzymes aspartate transaminase
ALT: Alanine transaminase
