# Supplementary material for: Efficacy and safety of traditional Chinese medicine for cancer-related fatigue: a systematic literature review of randomized controlled trials
Source: Chin Med. 2023 Nov 1;18:142. doi: 10.1186/s13020-023-00849-y (PMC10619240; doi:10.1186/s13020-023-00849-y)
Supplement: Supplementary file 1 — Additional file 1. A detailed description of each search strategy. [file 13020_2023_849_MOESM1_ESM.docx]

**A detailed description of each search strategy**

| **PubMed: search: 711** | | | | | |
| --- | --- | --- | --- | --- | --- |
| Search number | Query | Sort By | Filters | Search Details | Results |
| 37 | ((((fatigue[MeSH Terms]) OR (Lassitude[Title/Abstract])) OR (fatigue[Title/Abstract])) AND (((((((((((((((((Medicine, Traditional[MeSH Terms]) OR (Complementary Therapies[MeSH Terms])) OR (Phytotherapy[MeSH Terms])) OR (Plant Extracts[MeSH Terms])) OR (Plants, Medicinal[MeSH Terms])) OR (Plant Preparations[MeSH Terms])) OR (Drugs, Chinese Herbal[MeSH Terms])) OR (Medicine, Chinese Traditional[MeSH Terms])) OR ("complementary medicine*"[Title/Abstract])) OR ("alternative medicine*"[Title/Abstract])) OR ("Chinese medicine*"[Title/Abstract])) OR ("pharmaceutical plant*"[Title/Abstract])) OR ("medicinal plant*"[Title/Abstract])) OR (herb*[Title/Abstract])) OR (nutraceutical*[Title/Abstract])) OR ("folk remed*"[Title/Abstract])) OR ("folk medicine*"[Title/Abstract])) AND (randomizedcontrolledtrial[Filter])) NOT ((((massage[Title/Abstract]) OR (meditation[Title/Abstract])) OR (acupuncture[Title/Abstract])) OR (yoga[Title/Abstract]) AND (randomizedcontrolledtrial[Filter])) |  | Clinical Study, Clinical Trial, Clinical Trial, Phase I, Clinical Trial, Phase II, Clinical Trial, Phase III, Randomized Controlled Trial | ((("fatigue"[MeSH Terms] OR "Lassitude"[Title/Abstract] OR "fatigue"[Title/Abstract]) AND ("medicine, traditional"[MeSH Terms] OR "complementary therapies"[MeSH Terms] OR "phytotherapy"[MeSH Terms] OR "plant extracts"[MeSH Terms] OR "plants, medicinal"[MeSH Terms] OR "plant preparations"[MeSH Terms] OR "drugs, chinese herbal"[MeSH Terms] OR "medicine, chinese traditional"[MeSH Terms] OR "complementary medicine*"[Title/Abstract] OR "alternative medicine*"[Title/Abstract] OR "chinese medicine*"[Title/Abstract] OR "pharmaceutical plant*"[Title/Abstract] OR "medicinal plant*"[Title/Abstract] OR "herb*"[Title/Abstract] OR "nutraceutical*"[Title/Abstract] OR "folk remed*"[Title/Abstract] OR "folk medicine*"[Title/Abstract]) AND "randomized controlled trial"[Publication Type]) NOT (("massage"[Title/Abstract] OR "meditation"[Title/Abstract] OR "acupuncture"[Title/Abstract] OR "yoga"[Title/Abstract]) AND "randomized controlled trial"[Publication Type])) AND (clinicalstudy[Filter] OR clinicaltrial[Filter] OR clinicaltrialphasei[Filter] OR clinicaltrialphaseii[Filter] OR clinicaltrialphaseiii[Filter] OR randomizedcontrolledtrial[Filter]) | 711 |
| 30 | ((((fatigue[MeSH Terms]) OR (Lassitude[Title/Abstract])) OR (fatigue[Title/Abstract])) AND (((((((((((((((((Medicine, Traditional[MeSH Terms]) OR (Complementary Therapies[MeSH Terms])) OR (Phytotherapy[MeSH Terms])) OR (Plant Extracts[MeSH Terms])) OR (Plants, Medicinal[MeSH Terms])) OR (Plant Preparations[MeSH Terms])) OR (Drugs, Chinese Herbal[MeSH Terms])) OR (Medicine, Chinese Traditional[MeSH Terms])) OR ("complementary medicine*"[Title/Abstract])) OR ("alternative medicine*"[Title/Abstract])) OR ("Chinese medicine*"[Title/Abstract])) OR ("pharmaceutical plant*"[Title/Abstract])) OR ("medicinal plant*"[Title/Abstract])) OR (herb*[Title/Abstract])) OR (nutraceutical*[Title/Abstract])) OR ("folk remed*"[Title/Abstract])) OR ("folk medicine*"[Title/Abstract])) AND (randomizedcontrolledtrial[Filter])) NOT ((((massage[Title/Abstract]) OR (meditation[Title/Abstract])) OR (acupuncture[Title/Abstract])) OR (yoga[Title/Abstract]) AND (randomizedcontrolledtrial[Filter])) |  | Clinical Trial, Randomized Controlled Trial, in the last 10 years | ((("fatigue"[MeSH Terms] OR "Lassitude"[Title/Abstract] OR "fatigue"[Title/Abstract]) AND ("medicine, traditional"[MeSH Terms] OR "complementary therapies"[MeSH Terms] OR "phytotherapy"[MeSH Terms] OR "plant extracts"[MeSH Terms] OR "plants, medicinal"[MeSH Terms] OR "plant preparations"[MeSH Terms] OR "drugs, chinese herbal"[MeSH Terms] OR "medicine, chinese traditional"[MeSH Terms] OR "complementary medicine*"[Title/Abstract] OR "alternative medicine*"[Title/Abstract] OR "chinese medicine*"[Title/Abstract] OR "pharmaceutical plant*"[Title/Abstract] OR "medicinal plant*"[Title/Abstract] OR "herb*"[Title/Abstract] OR "nutraceutical*"[Title/Abstract] OR "folk remed*"[Title/Abstract] OR "folk medicine*"[Title/Abstract]) AND "randomized controlled trial"[Publication Type]) NOT (("massage"[Title/Abstract] OR "meditation"[Title/Abstract] OR "acupuncture"[Title/Abstract] OR "yoga"[Title/Abstract]) AND "randomized controlled trial"[Publication Type])) AND ((y_10[Filter]) AND (clinicaltrial[Filter] OR randomizedcontrolledtrial[Filter]) AND (2012:2022[pdat])) | 456 |
| 27 | ((((fatigue[MeSH Terms]) OR (Lassitude[Title/Abstract])) OR (fatigue[Title/Abstract])) AND (((((((((((((((((Medicine, Traditional[MeSH Terms]) OR (Complementary Therapies[MeSH Terms])) OR (Phytotherapy[MeSH Terms])) OR (Plant Extracts[MeSH Terms])) OR (Plants, Medicinal[MeSH Terms])) OR (Plant Preparations[MeSH Terms])) OR (Drugs, Chinese Herbal[MeSH Terms])) OR (Medicine, Chinese Traditional[MeSH Terms])) OR ("complementary medicine*"[Title/Abstract])) OR ("alternative medicine*"[Title/Abstract])) OR ("Chinese medicine*"[Title/Abstract])) OR ("pharmaceutical plant*"[Title/Abstract])) OR ("medicinal plant*"[Title/Abstract])) OR (herb*[Title/Abstract])) OR (nutraceutical*[Title/Abstract])) OR ("folk remed*"[Title/Abstract])) OR ("folk medicine*"[Title/Abstract])) AND (randomizedcontrolledtrial[Filter])) NOT ((((massage[Title/Abstract]) OR (meditation[Title/Abstract])) OR (acupuncture[Title/Abstract])) OR (yoga[Title/Abstract]) AND (randomizedcontrolledtrial[Filter])) |  | Randomized Controlled Trial, in the last 10 years | ((("fatigue"[MeSH Terms] OR "Lassitude"[Title/Abstract] OR "fatigue"[Title/Abstract]) AND ("medicine, traditional"[MeSH Terms] OR "complementary therapies"[MeSH Terms] OR "phytotherapy"[MeSH Terms] OR "plant extracts"[MeSH Terms] OR "plants, medicinal"[MeSH Terms] OR "plant preparations"[MeSH Terms] OR "drugs, chinese herbal"[MeSH Terms] OR "medicine, chinese traditional"[MeSH Terms] OR "complementary medicine*"[Title/Abstract] OR "alternative medicine*"[Title/Abstract] OR "chinese medicine*"[Title/Abstract] OR "pharmaceutical plant*"[Title/Abstract] OR "medicinal plant*"[Title/Abstract] OR "herb*"[Title/Abstract] OR "nutraceutical*"[Title/Abstract] OR "folk remed*"[Title/Abstract] OR "folk medicine*"[Title/Abstract]) AND "randomized controlled trial"[Publication Type]) NOT (("massage"[Title/Abstract] OR "meditation"[Title/Abstract] OR "acupuncture"[Title/Abstract] OR "yoga"[Title/Abstract]) AND "randomized controlled trial"[Publication Type])) AND ((y_10[Filter]) AND (randomizedcontrolledtrial[Filter])) | 456 |
| 26 | ((((fatigue[MeSH Terms]) OR (Lassitude[Title/Abstract])) OR (fatigue[Title/Abstract])) AND (((((((((((((((((Medicine, Traditional[MeSH Terms]) OR (Complementary Therapies[MeSH Terms])) OR (Phytotherapy[MeSH Terms])) OR (Plant Extracts[MeSH Terms])) OR (Plants, Medicinal[MeSH Terms])) OR (Plant Preparations[MeSH Terms])) OR (Drugs, Chinese Herbal[MeSH Terms])) OR (Medicine, Chinese Traditional[MeSH Terms])) OR ("complementary medicine*"[Title/Abstract])) OR ("alternative medicine*"[Title/Abstract])) OR ("Chinese medicine*"[Title/Abstract])) OR ("pharmaceutical plant*"[Title/Abstract])) OR ("medicinal plant*"[Title/Abstract])) OR (herb*[Title/Abstract])) OR (nutraceutical*[Title/Abstract])) OR ("folk remed*"[Title/Abstract])) OR ("folk medicine*"[Title/Abstract])) AND (randomizedcontrolledtrial[Filter])) NOT ((((massage[Title/Abstract]) OR (meditation[Title/Abstract])) OR (acupuncture[Title/Abstract])) OR (yoga[Title/Abstract]) AND (randomizedcontrolledtrial[Filter])) |  | Randomized Controlled Trial | ((("fatigue"[MeSH Terms] OR "Lassitude"[Title/Abstract] OR "fatigue"[Title/Abstract]) AND ("medicine, traditional"[MeSH Terms] OR "complementary therapies"[MeSH Terms] OR "phytotherapy"[MeSH Terms] OR "plant extracts"[MeSH Terms] OR "plants, medicinal"[MeSH Terms] OR "plant preparations"[MeSH Terms] OR "drugs, chinese herbal"[MeSH Terms] OR "medicine, chinese traditional"[MeSH Terms] OR "complementary medicine*"[Title/Abstract] OR "alternative medicine*"[Title/Abstract] OR "chinese medicine*"[Title/Abstract] OR "pharmaceutical plant*"[Title/Abstract] OR "medicinal plant*"[Title/Abstract] OR "herb*"[Title/Abstract] OR "nutraceutical*"[Title/Abstract] OR "folk remed*"[Title/Abstract] OR "folk medicine*"[Title/Abstract]) AND "randomized controlled trial"[Publication Type]) NOT (("massage"[Title/Abstract] OR "meditation"[Title/Abstract] OR "acupuncture"[Title/Abstract] OR "yoga"[Title/Abstract]) AND "randomized controlled trial"[Publication Type])) AND (randomizedcontrolledtrial[Filter]) | 711 |
| 25 | (((massage[Title/Abstract]) OR (meditation[Title/Abstract])) OR (acupuncture[Title/Abstract])) OR (yoga[Title/Abstract]) |  | Randomized Controlled Trial | ("massage"[Title/Abstract] OR "meditation"[Title/Abstract] OR "acupuncture"[Title/Abstract] OR "yoga"[Title/Abstract]) AND (randomizedcontrolledtrial[Filter]) | 6,510 |
| 24 | (((fatigue[MeSH Terms]) OR (Lassitude[Title/Abstract])) OR (fatigue[Title/Abstract])) AND (((((((((((((((((Medicine, Traditional[MeSH Terms]) OR (Complementary Therapies[MeSH Terms])) OR (Phytotherapy[MeSH Terms])) OR (Plant Extracts[MeSH Terms])) OR (Plants, Medicinal[MeSH Terms])) OR (Plant Preparations[MeSH Terms])) OR (Drugs, Chinese Herbal[MeSH Terms])) OR (Medicine, Chinese Traditional[MeSH Terms])) OR ("complementary medicine*"[Title/Abstract])) OR ("alternative medicine*"[Title/Abstract])) OR ("Chinese medicine*"[Title/Abstract])) OR ("pharmaceutical plant*"[Title/Abstract])) OR ("medicinal plant*"[Title/Abstract])) OR (herb*[Title/Abstract])) OR (nutraceutical*[Title/Abstract])) OR ("folk remed*"[Title/Abstract])) OR ("folk medicine*"[Title/Abstract])) |  | Randomized Controlled Trial | (("fatigue"[MeSH Terms] OR "Lassitude"[Title/Abstract] OR "fatigue"[Title/Abstract]) AND ("medicine, traditional"[MeSH Terms] OR "complementary therapies"[MeSH Terms] OR "phytotherapy"[MeSH Terms] OR "plant extracts"[MeSH Terms] OR "plants, medicinal"[MeSH Terms] OR "plant preparations"[MeSH Terms] OR "drugs, chinese herbal"[MeSH Terms] OR "medicine, chinese traditional"[MeSH Terms] OR "complementary medicine*"[Title/Abstract] OR "alternative medicine*"[Title/Abstract] OR "chinese medicine*"[Title/Abstract] OR "pharmaceutical plant*"[Title/Abstract] OR "medicinal plant*"[Title/Abstract] OR "herb*"[Title/Abstract] OR "nutraceutical*"[Title/Abstract] OR "folk remed*"[Title/Abstract] OR "folk medicine*"[Title/Abstract])) AND (randomizedcontrolledtrial[Filter]) | 986 |
| 23 | (((fatigue[MeSH Terms]) OR (Lassitude[Title/Abstract])) OR (fatigue[Title/Abstract])) AND (((((((((((((((((Medicine, Traditional[MeSH Terms]) OR (Complementary Therapies[MeSH Terms])) OR (Phytotherapy[MeSH Terms])) OR (Plant Extracts[MeSH Terms])) OR (Plants, Medicinal[MeSH Terms])) OR (Plant Preparations[MeSH Terms])) OR (Drugs, Chinese Herbal[MeSH Terms])) OR (Medicine, Chinese Traditional[MeSH Terms])) OR ("complementary medicine*"[Title/Abstract])) OR ("alternative medicine*"[Title/Abstract])) OR ("Chinese medicine*"[Title/Abstract])) OR ("pharmaceutical plant*"[Title/Abstract])) OR ("medicinal plant*"[Title/Abstract])) OR (herb*[Title/Abstract])) OR (nutraceutical*[Title/Abstract])) OR ("folk remed*"[Title/Abstract])) OR ("folk medicine*"[Title/Abstract])) |  |  | ("fatigue"[MeSH Terms] OR "Lassitude"[Title/Abstract] OR "fatigue"[Title/Abstract]) AND ("medicine, traditional"[MeSH Terms] OR "complementary therapies"[MeSH Terms] OR "phytotherapy"[MeSH Terms] OR "plant extracts"[MeSH Terms] OR "plants, medicinal"[MeSH Terms] OR "plant preparations"[MeSH Terms] OR "drugs, chinese herbal"[MeSH Terms] OR "medicine, chinese traditional"[MeSH Terms] OR "complementary medicine*"[Title/Abstract] OR "alternative medicine*"[Title/Abstract] OR "chinese medicine*"[Title/Abstract] OR "pharmaceutical plant*"[Title/Abstract] OR "medicinal plant*"[Title/Abstract] OR "herb*"[Title/Abstract] OR "nutraceutical*"[Title/Abstract] OR "folk remed*"[Title/Abstract] OR "folk medicine*"[Title/Abstract]) | 4,366 |
| 22 | ((((((((((((((((Medicine, Traditional[MeSH Terms]) OR (Complementary Therapies[MeSH Terms])) OR (Phytotherapy[MeSH Terms])) OR (Plant Extracts[MeSH Terms])) OR (Plants, Medicinal[MeSH Terms])) OR (Plant Preparations[MeSH Terms])) OR (Drugs, Chinese Herbal[MeSH Terms])) OR (Medicine, Chinese Traditional[MeSH Terms])) OR ("complementary medicine*"[Title/Abstract])) OR ("alternative medicine*"[Title/Abstract])) OR ("Chinese medicine*"[Title/Abstract])) OR ("pharmaceutical plant*"[Title/Abstract])) OR ("medicinal plant*"[Title/Abstract])) OR (herb*[Title/Abstract])) OR (nutraceutical*[Title/Abstract])) OR ("folk remed*"[Title/Abstract])) OR ("folk medicine*"[Title/Abstract]) |  |  | "medicine, traditional"[MeSH Terms] OR "complementary therapies"[MeSH Terms] OR "phytotherapy"[MeSH Terms] OR "plant extracts"[MeSH Terms] OR "plants, medicinal"[MeSH Terms] OR "plant preparations"[MeSH Terms] OR "drugs, chinese herbal"[MeSH Terms] OR "medicine, chinese traditional"[MeSH Terms] OR "complementary medicine*"[Title/Abstract] OR "alternative medicine*"[Title/Abstract] OR "chinese medicine*"[Title/Abstract] OR "pharmaceutical plant*"[Title/Abstract] OR "medicinal plant*"[Title/Abstract] OR "herb*"[Title/Abstract] OR "nutraceutical*"[Title/Abstract] OR "folk remed*"[Title/Abstract] OR "folk medicine*"[Title/Abstract] | 611,956 |
| 21 | "folk medicine*"[Title/Abstract] |  |  | "folk medicine*"[Title/Abstract] | 6,574 |
| 20 | "folk remed*"[Title/Abstract] |  |  | "folk remed*"[Title/Abstract] | 670 |
| 19 | nutraceutical*[Title/Abstract] |  |  | "nutraceutical*"[Title/Abstract] | 11,004 |
| 18 | herb*[Title/Abstract] |  |  | "herb*"[Title/Abstract] | 129,639 |
| 17 | "medicinal plant*"[Title/Abstract] |  |  | "medicinal plant*"[Title/Abstract] | 24,607 |
| 16 | "pharmaceutical plant*"[Title/Abstract] |  |  | "pharmaceutical plant*"[Title/Abstract] | 183 |
| 15 | "Chinese medicine*"[Title/Abstract] |  |  | "chinese medicine*"[Title/Abstract] | 36,529 |
| 14 | "alternative medicine*"[Title/Abstract] |  |  | "alternative medicine*"[Title/Abstract] | 11,794 |
| 13 | "complementary medicine*"[Title/Abstract] |  |  | "complementary medicine*"[Title/Abstract] | 4,614 |
| 12 | Medicine, Chinese Traditional[MeSH Terms] |  |  | "medicine, chinese traditional"[MeSH Terms] | 22,766 |
| 11 | Drugs, Chinese Herbal[MeSH Terms] |  |  | "drugs, chinese herbal"[MeSH Terms] | 50,590 |
| 10 | Plant Preparations[MeSH Terms] |  |  | "plant preparations"[MeSH Terms] | 258,689 |
| 9 | Plants, Medicinal[MeSH Terms] |  |  | "plants, medicinal"[MeSH Terms] | 61,836 |
| 8 | Plant Extracts[MeSH Terms] |  |  | "plant extracts"[MeSH Terms] | 197,086 |
| 7 | Phytotherapy[MeSH Terms] |  |  | "phytotherapy"[MeSH Terms] | 41,779 |
| 6 | Complementary Therapies[MeSH Terms] |  |  | "complementary therapies"[MeSH Terms] | 240,460 |
| 5 | Medicine, Traditional[MeSH Terms] |  |  | "medicine, traditional"[MeSH Terms] | 44,420 |
| 4 | ((fatigue[MeSH Terms]) OR (Lassitude[Title/Abstract])) OR (fatigue[Title/Abstract]) |  |  | "fatigue"[MeSH Terms] OR "Lassitude"[Title/Abstract] OR "fatigue"[Title/Abstract] | 126,340 |
| 3 | fatigue[Title/Abstract] |  |  | "fatigue"[Title/Abstract] | 116,403 |
| 2 | Lassitude[Title/Abstract] |  |  | "Lassitude"[Title/Abstract] | 459 |
| 1 | fatigue[MeSH Terms] |  |  | "fatigue"[MeSH Terms] | 35,703 |

| **Scopus: search: 927** | | |
| --- | --- | --- |
| Search number | Query | Results |
| 11 | ( ( ( TITLE-ABS-KEY ( fatigue ) ) OR ( TITLE-ABS-KEY ( lassitude ) ) ) AND ( ( TITLE-ABS-KEY ( "traditional medicine*" OR "complementary medicine*" OR "complementary therap*" OR "phytotherapy" OR "plant extract*" OR "herb*" OR "chinese medicine*" OR "alternative medicine*" OR "pharmaceutical plant*" OR "medicinal plant*" OR "nutraceutical*" OR "folk" ) ) AND NOT ( TITLE-ABS-KEY ( "massage" OR "yoga" OR "acupuncture" OR "music" OR "meditation" OR "hypnosis" OR "exercise" OR "moxibustion" OR "acupressure" OR "aromatherapy " OR "muscle" OR "manipulat*" OR "mindful" OR "relaxation" ) ) ) ) AND ( TITLE-ABS-KEY ( "clinical trial*" OR "clinical stud*" ) ) AND ( LIMIT-TO ( DOCTYPE , "ar" ) ) AND ( EXCLUDE ( EXACTKEYWORD , "pilot study" ) OR EXCLUDE ( EXACTKEYWORD , "questionnaires" ) OR EXCLUDE ( EXACTKEYWORD , "cross-sectional study" ) OR EXCLUDE ( EXACTKEYWORD , "surveys and questionnaires" ) OR EXCLUDE ( EXACTKEYWORD , "observational study" ) ) | 927 |
| 10 | ( ( ( TITLE-ABS-KEY ( fatigue ) ) OR ( TITLE-ABS-KEY ( lassitude ) ) ) AND ( ( TITLE-ABS-KEY ( "traditional medicine*" OR "complementary medicine*" OR "complementary therap*" OR "phytotherapy" OR "plant extract*" OR "herb*" OR "chinese medicine*" OR "alternative medicine*" OR "pharmaceutical plant*" OR "medicinal plant*" OR "nutraceutical*" OR "folk" ) ) AND NOT ( TITLE-ABS-KEY ( "massage" OR "yoga" OR "acupuncture" OR "music" OR "meditation" OR "hypnosis" OR "exercise" OR "moxibustion" OR "acupressure" OR "aromatherapy " OR "muscle" OR "manipulat*" OR "mindful" OR "relaxation" ) ) ) ) AND ( TITLE-ABS-KEY ( "clinical trial*" OR "clinical stud*" ) ) AND ( LIMIT-TO ( DOCTYPE , "ar" ) ) | 1,177 |
| 9 | ( ( ( TITLE-ABS-KEY ( fatigue ) ) OR ( TITLE-ABS-KEY ( lassitude ) ) ) AND ( ( TITLE-ABS-KEY ( "traditional medicine*" OR "complementary medicine*" OR "complementary therap*" OR "phytotherapy" OR "plant extract*" OR "herb*" OR "chinese medicine*" OR "alternative medicine*" OR "pharmaceutical plant*" OR "medicinal plant*" OR "nutraceutical*" OR "folk" ) ) AND NOT ( TITLE-ABS-KEY ( "massage" OR "yoga" OR "acupuncture" OR "music" OR "meditation" OR "hypnosis" OR "exercise" OR "moxibustion" OR "acupressure" OR "aromatherapy " OR "muscle" OR "manipulat*" OR "mindful" OR "relaxation" ) ) ) ) AND ( TITLE-ABS-KEY ( "clinical trial*" OR "clinical stud*" ) ) | 1,629 |
| 8 | TITLE-ABS-KEY ( "clinical trial*" OR "clinical stud*" ) | 5,051,598 |
| 7 | ( ( TITLE-ABS-KEY ( fatigue ) ) OR ( TITLE-ABS-KEY ( lassitude ) ) ) AND ( ( TITLE-ABS-KEY ( "traditional medicine*" OR "complementary medicine*" OR "complementary therap*" OR "phytotherapy" OR "plant extract*" OR "herb*" OR "chinese medicine*" OR "alternative medicine*" OR "pharmaceutical plant*" OR "medicinal plant*" OR "nutraceutical*" OR "folk" ) ) AND NOT ( TITLE-ABS-KEY ( "massage" OR "yoga" OR "acupuncture" OR "music" OR "meditation" OR "hypnosis" OR "exercise" OR "moxibustion" OR "acupressure" OR "aromatherapy " OR "muscle" OR "manipulat*" OR "mindful" OR "relaxation" ) ) ) | 3,997 |
| 6 | ( TITLE-ABS-KEY ( "traditional medicine*" OR "complementary medicine*" OR "complementary therap*" OR "phytotherapy" OR "plant extract*" OR "herb*" OR "chinese medicine*" OR "alternative medicine*" OR "pharmaceutical plant*" OR "medicinal plant*" OR "nutraceutical*" OR "folk" ) ) AND NOT ( TITLE-ABS-KEY ( "massage" OR "yoga" OR "acupuncture" OR "music" OR "meditation" OR "hypnosis" OR "exercise" OR "moxibustion" OR "acupressure" OR "aromatherapy " OR "muscle" OR "manipulat*" OR "mindful" OR "relaxation" ) ) | 777,107 |
| 5 | TITLE-ABS-KEY ( "massage" OR "yoga" OR "acupuncture" OR "music" OR "meditation" OR "hypnosis" OR "exercise" OR "moxibustion" OR "acupressure" OR "aromatherapy " OR "muscle" OR "manipulat*" OR "mindful" OR "relaxation" ) | 3,653,250 |
| 4 | TITLE-ABS-KEY ( "traditional medicine*" OR "complementary medicine*" OR "complementary therap*" OR "phytotherapy" OR "plant extract*" OR "herb*" OR "chinese medicine*" OR "alternative medicine*" OR "pharmaceutical plant*" OR "medicinal plant*" OR "nutraceutical*" OR "folk" ) | 835,498 |
| 3 | ( TITLE-ABS-KEY ( fatigue ) ) OR ( TITLE-ABS-KEY ( lassitude ) ) | 507,372 |
| 2 | TITLE-ABS-KEY ( lassitude ) | 1,237 |
| 1 | TITLE-ABS-KEY ( fatigue ) | 506,419 |

| **Web of Science: search: 213** | | |
| --- | --- | --- |
| Search number | Query | Results |
| 10 | #8 AND #9 | 213 |
| 9 | TS=("clinical trial*" OR "clinical stud*") | 622,737 |
| 8 | #3 AND #6 and Article (Document Types) | 1,309 |
| 7 | #3 AND #6 | 1,773 |
| 6 | #4 NOT #5 | 445,351 |
| 5 | TI=( "massage" OR "yoga" OR "acupuncture" OR "music" OR "meditation" OR "hypnosis" OR "exercise" OR "moxibustion" OR "acupressure" OR "aromatherapy " OR "muscle" OR "manipulat*" OR "mindful" OR "relaxation") | 745,366 |
| 4 | TS=( "traditional medicine*" OR "complementary medicine*" OR "complementary therap*" OR "phytotherapy" OR "plant extract*" OR "herb*" OR "chinese medicine*" OR "alternative medicine*" OR "pharmaceutical plant*" OR "medicinal plant*" OR "nutraceutical*" OR "folk") | 452,939 |
| 3 | #1 OR #2 | 288,651 |
| 2 | TS=(lassitude) | 313 |
| 1 | TS=(fatigue) | 288,406 |

| **Embase: search: 444** | | |
| --- | --- | --- |
| No. | Query | Results |
| #54 | #40 NOT #53 | 444 |
| #53 | #45 OR #46 OR #47 OR #48 OR #49 OR #50 OR #51 OR #52 | 1317254 |
| #52 | observational':ti | 60887 |
| #51 | retrospective':ti | 160017 |
| #50 | questionnaire*':ti | 49381 |
| #49 | cross-sectional study':ti | 55169 |
| #48 | meta analysis:ti | 188735 |
| #47 | review:ti | 738624 |
| #46 | pilot:ti | 118849 |
| #45 | protocol:ti | 84134 |
| #44 | #40 AND #43 | 140 |
| #43 | #41 OR #42 | 878245 |
| #42 | 'clinical stud*':ab,ti,kw | 239637 |
| #41 | 'clinical trial*':ab,ti,kw | 666422 |
| #40 | #37 AND [humans]/lim AND [clinical study]/lim AND [article]/lim AND ([chinese]/lim OR [english]/lim) | 550 |
| #39 | #37 AND [humans]/lim AND [clinical study]/lim AND [article]/lim | 576 |
| #38 | #37 AND [humans]/lim AND [clinical study]/lim | 831 |
| #37 | #21 NOT #36 | 1759 |
| #36 | #22 OR #23 OR #24 OR #25 OR #26 OR #27 OR #28 OR #29 OR #30 OR #31 OR #32 OR #33 OR #34 OR #35 | 1713750 |
| #35 | 'relaxation':ab,ti,kw | 145815 |
| #34 | 'mindful':ab,ti,kw | 5976 |
| #33 | 'manipulat*':ab,ti,kw | 253998 |
| #32 | 'muscle':ab,ti,kw | 917563 |
| #31 | 'aromatherapy':ab,ti,kw | 2311 |
| #30 | 'acupressure':ab,ti,kw | 1934 |
| #29 | 'moxibustion':ab,ti,kw | 4482 |
| #28 | 'exercise':ab,ti,kw | 418644 |
| #27 | 'hypnosis':ab,ti,kw | 10549 |
| #26 | 'meditation':ab,ti,kw | 8791 |
| #25 | 'music':ab,ti,kw | 25375 |
| #24 | 'acupuncture':ab,ti,kw | 37467 |
| #23 | 'yoga':ab,ti,kw | 8982 |
| #22 | 'massage':ab,ti,kw | 16038 |
| #21 | #7 AND #20 | 2661 |
| #20 | #8 OR #9 OR #10 OR #11 OR #12 OR #13 OR #14 OR #15 OR #16 OR #17 OR #18 OR #19 | 311567 |
| #19 | 'folk':ab,ti,kw | 14880 |
| #18 | 'nutraceutical*':ab,ti,kw | 12851 |
| #17 | 'medicinal plant*':ab,ti,kw | 42383 |
| #16 | 'pharmaceutical plant*':ab,ti,kw | 284 |
| #15 | 'alternative medicine*':ab,ti,kw | 16938 |
| #14 | 'chinese medicine*':ab,ti,kw | 49043 |
| #13 | 'herb*':ab,ti,kw | 166166 |
| #12 | 'plant extract*':ab,ti,kw | 21731 |
| #11 | 'phytotherapy':ab,ti,kw | 4342 |
| #10 | 'complementary therap*':ab,ti,kw | 7200 |
| #9 | 'complementary medicine*':ab,ti,kw | 7177 |
| #8 | 'traditional medicine*':ab,ti,kw | 24891 |
| #7 | #5 OR #6 | 189679 |
| #6 | lassitude:ab,ti,kw | 661 |
| #5 | fatigue:ab,ti,kw | 189142 |
| #4 | #1 OR #2 | 324795 |
| #3 | 'traditional medicine*' OR 'complementary medicine*' OR 'complementary therap*' OR 'phytotherapy'/exp OR 'phytotherapy' OR 'plant extract*' OR 'herb*' OR 'chinese medicine*' OR 'alternative medicine*' OR 'pharmaceutical plant*' OR 'medicinal plant*' OR 'nutraceutical*' OR 'folk' | 783135 |
| #2 | 'fatigue' | 323852 |
| #1 | lassitude | 1234 |

| **Cochrane: search: 476** | | |
| --- | --- | --- |
| ID | Search | Hits |
| #1 | MeSH descriptor: [Fatigue] explode all trees | 4303 |
| #2 | Lassitude' in Trials | 159 |
| #3 | MeSH descriptor: [Medicine, Traditional] explode all trees | 1644 |
| #4 | MeSH descriptor: [Complementary Therapies] explode all trees | 22190 |
| #5 | MeSH descriptor: [Phytotherapy] explode all trees | 4329 |
| #6 | MeSH descriptor: [Plant Extracts] explode all trees | 8917 |
| #7 | MeSH descriptor: [Plant Preparations] explode all trees | 12476 |
| #8 | MeSH descriptor: [Drugs, Chinese Herbal] explode all trees | 3808 |
| #9 | MeSH descriptor: [Medicine, Chinese Traditional] explode all trees | 1276 |
| #10 | #1 OR #2 | 4457 |
| #11 | #3 OR #4 OR #5 OR #6 OR #7 OR #8 OR #9 | 30551 |
| #12 | #10 AND #11 in Trials | 476 |

| **CNKI: search: 460** | | | | |
| --- | --- | --- | --- | --- |
|  | 檢索條件 | 檢索範圍 | 數目 | 檢索時間 |
| 1 | 篇關摘（疲勞 + 慢性疲勞 + 慢性疲勞綜合徵 + 肌痛性脊髓炎 + 慢性疲勞免疫功能障礙綜合症 + 疲勞綜合症 + 特發性慢性疲勞 + 癌因性疲勞）AND 篇關摘（隨機 + 對照 + 隨機對照）AND 篇關摘（實驗 + 臨床實驗 + 臨床研究 + 臨床試驗 + 臨床觀察） AND 篇關摘（中藥 + 中醫藥 + 中草藥）NOT 篇關摘（針灸 + 針刺 + 熏蒸 + 推拿 + 穴 + 音樂 + 氣 + 經驗 + 針 + 功） | 資源範圍:會議；中英文擴展；更新時間:不限；會議級別:全部,報告級別:全部,論文集類型:不限,語種:不限 | 31 | 2022/12/10 |
| 2 | 篇關摘（疲勞 + 慢性疲勞 + 慢性疲勞綜合徵 + 肌痛性脊髓炎 + 慢性疲勞免疫功能障礙綜合症 + 疲勞綜合症 + 特發性慢性疲勞 + 癌因性疲勞）AND 篇關摘（隨機 + 對照 + 隨機對照）AND 篇關摘（實驗 + 臨床實驗 + 臨床研究 + 臨床試驗 + 臨床觀察） AND 篇關摘（中藥 + 中醫藥 + 中草藥）NOT 篇關摘（針灸 + 針刺 + 熏蒸 + 推拿 + 穴 + 音樂 + 氣 + 經驗 + 針 + 功） | 資源範圍:學術期刊；中英文擴展；更新時間:不限；來源類別：全部期刊 | 249 | 2022/12/11 |
| 3 | 篇關摘（癌因性疲勞 + 癌相關性疲勞 + 腫瘤相關性疲勞 + 癌因性疲乏 + 癌性疲勞 + 癌性疲乏 + 腫瘤相關性疲乏）AND 篇關摘（隨機 + 對照 + 隨機對照）AND 篇關摘（實驗 + 臨床實驗 + 臨床研究 + 臨床試驗 + 臨床觀察）AND 篇關摘（中藥 + 中醫藥 + 中草藥）NOT 篇關摘（針灸 + 針刺 + 熏蒸 + 推拿 + 穴 + 音樂 + 氣 + 經驗 + 針 + 功） | 資源範圍:會議；中英文擴展；更新時間:不限；會議級別:全部,報告級別:全部,論文集類型:不限,語種:不限 | 2 | 2022/12/24 |
| 4 | 篇關摘（癌因性疲勞 + 癌相關性疲勞 + 腫瘤相關性疲勞 + 癌因性疲乏 + 癌性疲勞 + 癌性疲乏 + 腫瘤相關性疲乏）AND 篇關摘（隨機 + 對照 + 隨機對照）AND 篇關摘（實驗 + 臨床實驗 + 臨床研究 + 臨床試驗 + 臨床觀察）AND 篇關摘（中藥 + 中醫藥 + 中草藥）NOT 篇關摘（針灸 + 針刺 + 熏蒸 + 推拿 + 穴 + 音樂 + 氣 + 經驗 + 針 + 功） | 資源範圍:學術期刊；中英文擴展；更新時間:不限；來源類別：全部期刊 | 30 | 2022/12/24 |
| 5 | 主題（癌因性疲乏的臨床觀察） | 資源範圍:學術期刊 | 109 | 2022/12/28 |
| 6 | 從其他參考文獻中找出 |  | 39 |  |

| **Wanfang: search: 107** | | | | |
| --- | --- | --- | --- | --- |
|  | 文獻類型 | 檢索式 | 檢索結果 | 檢索時間 |
| 1 | 期刊、會議 | 主題:(“疲勞” or “慢性疲勞”or “慢性疲勞綜合徵” or “肌痛性脊髓炎” or “慢性疲勞免疫功能障礙綜合症” or “疲勞綜合症” or “特發性慢性疲勞” or “癌因性疲勞") and 主題:("隨機” or “對照” or “隨機對照") and 主題:("實驗” or “臨床實驗” or “臨床研究” or “臨床試驗” or “臨床觀察") and 主題:("中藥” or “中醫藥” or “中草藥") not 主題:("針灸” or “針刺” or “熏蒸” or “推拿” or “穴” or “音樂” or “氣” or “經驗” or “針” or “功") | 98 | 2022/12/12 |
| 2 | 期刊、會議 | 主題:(“癌因性疲勞” or “癌相關性疲勞” or “腫瘤相關性疲勞” or “癌因性疲乏” or “癌性疲乏” or “腫瘤相關性疲乏”or “癌性疲乏”) and 主題:("隨機” or “對照” or “隨機對照") and 主題:("實驗” or “臨床實驗” or “臨床研究” or “臨床試驗” or “臨床觀察") and 主題:("中藥” or “中醫藥” or “中草藥") not 主題:("針灸” or “針刺” or “熏蒸” or “推拿” or “穴” or “音樂” or “氣” or “經驗” or “針” or “功") | 8 | 2022/12/23 |
| 3 | 從其他參考文獻中找出 |  | 1 |  |
