# Supplementary material for: Efficacy and safety of traditional Chinese medicine for cancer-related fatigue: a systematic literature review of randomized controlled trials
Source: Chin Med. 2023 Nov 1;18:142. doi: 10.1186/s13020-023-00849-y (PMC10619240; doi:10.1186/s13020-023-00849-y)
Supplement: Supplementary file 2 — Additional file 2: Table S1. Level of risks of bias. Table S2. Diagnosis and included criteria of the CRF participants in RCTs in this review. Table S3. Evaluation of included trial studies using the CONSORT-CHM statement. Figure S1a. Risk of bias summary: review authors' judgements about each risk of bias item for each included study (Study No. 01-42). Figure S1b. Risk of bias summary: review authors' judgements about each risk of bias item for each included study (Study No. 43-81). [file 13020_2023_849_MOESM2_ESM.docx]

| **Additional document 2. Table S1. Level of risks of bias** | | | |
| --- | --- | --- | --- |
| **Risk items** | **Low risk of bias** | **Unclear risk of bias** | **High risk of bias** |
| 1. Sequence generation | Random methods used were specific described (e.g., table of random numbers, or computer random number generation, coin toss) | Information about sequence generation was unknown or difficult to determine whether it was “low risk” or “high risk” | A non-random method in the sequence generation process was indicated (e.g., hospitalization or medical treatment number, odd or even number of birthday) |
| 1. Allocation concealment | Participants and the researchers who recruited subjects were not able to predict the allocation (e.g., center assignment, identical containers, opaque sealed envelopes) | No specified information about the allocation methods. Insufficient information to determine “low risk” or “high risk”. | Participants or the researchers who recruited subjects could predict the allocation (e.g., data of birth, case number, opened envelopes) |
| 1. Blinding of participants and personnel | No blinding or imperfect blinding, but the system evaluators judged that the outcome would not be affected by the unblinding method, or participants and the researchers were blinded, and the blinding might not be broken. | Blinding and the methods in the study were not mentioned or insufficient descriptions. | Blinding was not used or was incomplete, the result judgment or measurement would be affected, or blinding of subjects and researchers, but the blinding might be broken |
| 1. Blinding of outcome assessment | Outcome evaluators were blinded, and the blinding might not be broken. | Blinding and the methods in the study were not mentioned or insufficient descriptions. | Blinding was not used or was incomplete, the result judgment or measurement would be affected, or blinding of outcome evaluators, but the blinding might be broken |
| 1. Incomplete outcome data | There were no missing data in the study, or the missing data did not affect the analysis of results | Information was incomplete, and it was difficult to judge whether the data was complete (e.g., number of missing persons or reasons not reported), or the study did not address issues of completeness | The number of participants and causes of absence between groups in the studies was unbalanced or inappropriate methods for dealing with missing data |
| 1. Selective outcome reporting | A study proposal or all desired outcomes in published studies | The information was incomplete and it was difficult to judge whether there was a risk of selective reporting of results | All pre-specified primary outcome measures were not reported, or important outcomes were not reported in the studies |
| 1. Other bias | No other sources of bias in the studies | There was insufficient information to determine whether there was an important risk of bias, or there was no good reason or evidence that could lead to bias | There were potential biases related to particular study designs in the studies, or the trial claims were falsified |

| **Additional document 2. Table S2: Diagnosis and included criteria of the CRF participants in RCTs in this review** | | | | | | | | |
| --- | --- | --- | --- | --- | --- | --- | --- | --- |
| No. | Author, year | ICD-10 | Scale | Chinese medicine diagnosis | Tumor diagnosis | Cancer classification | Other tests | Life expectancy |
| 1 | (Gu et al, 2021) | Yes | N/A | Deficiency of spleen and kidney | Yes | Stomach, colon | N/A | >6m |
| 2 | (Li et al, 2020) | Yes | PFS | *Qi* deficiency of lung and spleen | Yes | Lung, stomach | N/A | ≥3m |
| 3 | (Lin et al, 2020) | N/A | PFS>3, KPS>60 | *Qi*, blood, and *yin* deficiency | Yes | Others | N/A | >3m |
| 4 | (Hu et al, 2020) | N/A | N/A | N/A | N/A | Breast | N/A | N/A |
| 5 | (Wang et al, 2016) | Yes | KPS>50 | N/A | Yes | Breast | N/A | ≥6m |
| 6 | (Zhao et al, 2011) | N/A | Yes | N/A | Yes | Breast | N/A | N/A |
| 7 | (Cao, 2020) | Yes | KPS ≥70 | *Qi* and blood deficiency | Yes | Lung, breast, stomach, ovarian, colorectum, ureteral, diffuse large B cell lymphoma | N/A | >3m |
| 8 | (Ou et al, 2022) | Yes | KPS ≥60 | *Qi* and blood deficiency | Yes | Liver | N/A | ≥3m |
| 9 | (Zhang et al, 2018) | N/A | NCCN NGFRS | N/A | Yes | Lung; breast; stomach; ovarian; bowel | N/A | >6m |
| 10 | (Zhu et al, 2016) | N/A | KPS ≥40 | Liver and stomach disharmony, *qi* stagnation and blood stasis, accumulation of phlegm and dampness, *yang* deficiency of spleen and kidney, damp heat and *yin* deficiency | Yes | Stomach | N/A | N/A |
| 11 | (Wang et al, 2015) | Yes | ECOG-PS≤2 | *Yin* deficiency and internal heat, spleen deficiency and phlegm dampness, *qi* and *yin* deficiency, *yin* and *yang* deficiency, stagnation of *qi* and blood fatigue | Yes | Lung | N/A | ≥3m |
| 12 | (Chen, 2011) | N/A | BFI | N/A | N/A | Breast | N/A | N/A |
| 13 | (Yang et al, 2015) | Yes | KPS ≥60 | N/A | Yes | Lung; breast; stomach; ovarian; bowel; pancreatic | N/A | >3m |
| 14 | (Kong et al, 2016) | N/A | N/A | N/A | Yes | Lung | N/A | ≥6m |
| 15 | (Su et al, 2022) | N/A | PFS>3, KPS＞50 | Liver depression and spleen deficiency | Yes | Breast | N/A | >3m |
| 16 | (Jiang, 2022) | N/A | N/A | N/A | Yes | Breast; esophageal; bowel | N/A | >3m |
| 17 | (Luo et al, 2021) | Yes | KPS ≥50 | *Qi* deficiency of lung and spleen | Yes | Lung; breast; stomach; bowel; kidney; gynecological | N/A | >3m |
| 18 | (Wang et al, 2021) | N/A | KPS ≥60 | N/A | Yes | Stomach; liver; esophageal; colon; pancreatic; gallbladder | N/A | >3m |
| 19 | (He et al, 2020) | Yes | KPS ≥60 | Deficiency of spleen and kidney | Yes | Bowel | N/A | N/A |
| 20 | (Luo et al, 2019) | Yes | N/A | N/A | Yes | Lung; digestive tract | N/A | N/A |
| 21 | (Liu, 2018) | Yes | N/A | N/A | Yes | Digestive tract | N/A | N/A |
| 22 | (Lin et al, 2018) | N/A | N/A | N/A | Yes | Lung | N/A | >3m |
| 23 | (Li, 2016) | Yes | KPS ≥50 | N/A | Yes | Colorectum | N/A | ≥3m |
| 24 | (Song et al, 2016) | Yes | KPS >50 | N/A | Yes | Lung; breast; liver; cervical; rectal | N/A | >30d |
| 25 | (Liu et al, 2016) | N/A | N/A | N/A | Yes | Lung; breast; rectal; colon | HGB＞80 g /L | >3m |
| 26 | (Li, 2016) | Yes | KPS ≥50 | Deficiency of spleen and kidney | Yes | Bowel | N/A | ≥3m |
| 27 | (Zhang et al, 2016) | Yes | PFS>3, KPS ≥50 | N/A | Yes | Lung | N/A | >3m |
| 28 | (Liang et al, 2016) | N/A | N/A | N/A | Yes | Lung; breast; cervical; colorectum; nasopharyngeal carcinoma | N/A | ≥3m |
| 29 | (Sun, 2015) | N/A | KPS >60 | N/A | Yes | Stomach; esophageal; colorectum | N/A | >3m |
| 30 | (Li et al, 2011) | N/A | KPS ≥60 | N/A | Yes | Lung | N/A | ≥6m |
| 31 | (Huang, 2001) | N/A | QLQ-C30: (very fatigue or severe fatigue),  KPS: 20-50 | N/A | Yes | Lung; breast; stomach; liver; ovarian; cervical; esophageal; colorectum; laryngeal; bladder; prostate; malignant lymphoma; nasopharyngeal carcinoma | N/A | N/A |
| 32 | (Cao et al, 2022) | Yes | ECOG-PS ≤2 | *Qi* deficiency | Yes | Lung; breast; stomach; bowel; kidney; nasopharyngeal carcinoma | N/A | ≥6m |
| 33 | (Zhang et al, 2019) | Yes | KPS ≥50 | N/A | Yes | Others | N/A | ≥3m |
| 34 | (Wang, 2019) | N/A | N/A | N/A | N/A | Breast | N/A | N/A |
| 35 | (Yao, 2019) | N/A | N/A | N/A | Yes | Lung | N/A | >3m |
| 36 | (Chen et al, 2019) | Yes | KPS >70 | *Qi* and blood deficiency | N/A | Stomach | N/A | ≥3m |
| 37 | (Wang, 2018) | N/A | N/A | N/A | Yes | Stomach | N/A | N/A |
| 38 | (Guo et al, 2017) | Yes | KPS ≥60 | *Qi* deficiency | Yes | Lung | N/A | >3m |
| 39 | (Gu et al, 2009) | N/A | KPS >60 | N/A | Yes | Breast; stomach; rectal; colon | N/A | >6m |
| 40 | (Liu et al, 2014) | Yes | KPS >70 | N/A | Yes | Colorectal | N/A | N/A |
| 41 | (Li et al, 2013) | Yes | N/A | N/A | Yes | Lung; breast; stomach; ovarian; bowel; kidney; nasopharyngeal carcinoma | N/A | ≥3m |
| 42 | (Wang et al, 2016) | Yes | KPS >50 | N/A | Yes | Colorectum | N/A | >4m |
| 43 | (Cai, 2016) | N/A | N/A | *Qi* deficiency of spleen | Yes | Lung; breast; stomach; liver; esophageal; bowel; pancreatic; gallbladder; nasopharyngeal carcinoma | N/A | N/A |
| 44 | (Ning et al, 2020) | Yes | KPS >60 | *Qi* deficiency of spleen | Yes | Lung | N/A | >3m |
| 45 | (Yang, 2020) | N/A | N/A | N/A | Yes | Lung | N/A | N/A |
| 46 | (Shan et al, 2020) | N/A | N/A | N/A | Yes | Lung | N/A | >3m |
| 47 | (Wu, 2018) | N/A | N/A | N/A | N/A | Lung | N/A | N/A |
| 48 | (Shi, 2017) | N/A | KPS >60 | N/A | N/A | Lung | N/A | >6m |
| 49 | (Zhang et al, 2017) | N/A | N/A | N/A | Yes | Lung | N/A | >6m |
| 50 | (Wang et al, 2015) | Yes | KPS >60 | N/A | Yes | Lung | N/A | >3m |
| 51 | (Lu, 2014) | N/A | KPS >60 | N/A | Yes | Lung; breast; stomach; colorectum; others | N/A | >6m |
| 52 | (Zhang et al, 2019) | N/A | N/A | N/A | N/A | Gastrointestinal | N/A | N/A |
| 53 | (Zhang et al, 2017) | Yes | N/A | N/A | Yes | Colorectum | N/A | ≥3m |
| 54 | (Wu et al, 2014) | N/A | N/A | N/A | Yes | Lung | N/A | N/A |
| 55 | (Feng, 2014) | N/A | N/A | N/A | N/A | Lung | N/A | N/A |
| 56 | (Zhang et al, 2012) | N/A | KPS >60 | N/A | Yes | Pancreatic | N/A | >3m |
| 57 | (Li, 2015) | Yes | KPS ≥50 | Deficiency of spleen and kidney | Yes | Colorectum | N/A | ≥3m |
| 58 | (Yang et al, 2018) | N/A | N/A | N/A | Yes | Lung; stomach; esophageal; rectal; nasopharyngeal carcinoma | N/A | >3m |
| 59 | (Ou et al, 2016) | N/A | N/A | N/A | N/A | Lung | N/A | N/A |
| 60 | (Zhao, 2015) | N/A | N/A | N/A | Yes | Lung | N/A | N/A |
| 61 | (Leng, 2015) | N/A | KPS >60 | N/A | Yes | Lung | N/A | >3m |
| 62 | (Jing et al, 2010) | N/A | KPS >60 | N/A | Yes | Lung | N/A | >3m |
| 63 | (Huang, 2012) | N/A | KPS ≥60 | N/A | Yes | Lung | N/A | ≥3m |
| 64 | (Wei, 2016) | N/A | N/A | N/A | Yes | Lung | N/A | N/A |
| 65 | (Wu et al, 2014) | N/A | N/A | N/A | Yes | Lung | N/A | >6m |
| 66 | (Dai et al, 2013) | N/A | KPS >60 | N/A | Yes | Lung | N/A | N/A |
| 67 | (Huang et al, 2013) | N/A | ECOG: 0~2 | N/A | Yes | Lung; breast; esophageal; colorectum; nasopharyngeal carcinoma | N/A | 3m |
| 68 | (Wu, 2014) | N/A | KPS >60 | N/A | Yes | Lung; breast; stomach; liver; esophageal; colorectum; nasopharyngeal carcinoma | N/A | >6m |
| 69 | (Liang et al, 2012) | Yes | KPS >60 | *Qi* and *yin* deficiency | Yes | Lung; breast; stomach; liver; colorectum; others | N/A | >6m |
| 70 | (Wei, 2021) | N/A | N/A | N/A | N/A | Others | N/A | N/A |
| 71 | (Cui et al, 2022) | Yes | ECOG: 0~1 | *Qi* deficiency | Yes | Breast | N/A | >9m |
| 72 | (Guglielmo et al, 2020) | N/A | BFI >4 | N/A | Yes | head and neck | N/A | N/A |
| 73 | (Jeong et al, 2010) | N/A | VAS>40mm, ECOG ≤2 | N/A | Yes | Lung; breast; stomach; colon; others | N/A | N/A |
| 74 | (Gu et al, 2010) | N/A | N/A | N/A | Yes | Lung; breast; stomach; liver; colorectum; pancreatic | N/A | N/A |
| 75 | (Oliveira et al, 2011) | N/A | change in BFI | N/A | Yes | Breast | N/A | N/A |
| 76 | (Kim et al, 2020) | N/A | ECOG: 0~1 | N/A | Yes | Colorectum | AST, ALT≤2.5X ULN, Cr≤1.5X ULN, Hb≥9 g/dL | >6m |
| 77 | (Barton et al, 2010) | N/A | a question about fatigue level ≥4 | N/A | Yes | Lung; breast; colon; others | fatigue for at least 1 month | ≥6m |
| 78 | (Sette et al, 2018) a | N/A | increase in BFI | N/A | Yes | Breast | N/A | N/A |
|  | (Sette et al, 2018) b | N/A | increase in BFI and CFS* | N/A | Yes | Breast | N/A | N/A |
| 79 | (Lee et al, 2021) | N/A | BFI≥4, ECOG≤2 | N/A | Yes | Lung; breast; gastrointestinal; head and neck; urogenital | fatigue for at least 1 month | N/A |
| 80 | (Barton et al, 2013) | N/A | 11-point fatigue scale ≥4 | N/A | Yes | Breast; colon; prostate; hematologic; Hematologic; combination/unknown/other | fatigue for at least 1 month | N/A |
| 81 | (Costa et al, 2009) | N/A | N/A | N/A | Yes | Breast | N/A | N/A |

Notes: ICD-10 (The International Statistical Classification of Diseases and Related Health Problems 10th Revision)

Scale: PFS (Piper Fatigue Scale), KPS (Karnofsky Performance Status), ECOG (Eastern cooperative Oncology Group Scale), BFI (Brief Fatigue Inventory Scale), QLQ-C30 (EORTC Quality of Life Questionnaires), NGFRS (NCCN Guidelines Fatigue Rating Scale), CFS* (Chalder Fatigue Scale), VAS (Visual Analogue Scale)

Other tests: HGB (Hemoglobin), AST (Enzymes Aspartate Transaminase), ALT (Alanine Transaminase), Hb (hemoglobin)

| **Additional document 2. Table S3. Evaluation of included trial studies using the CONSORT-CHM statement** | | | | | | | | | | | | | | | | | | | | | | | | | | | | | |
| --- | --- | --- | --- | --- | --- | --- | --- | --- | --- | --- | --- | --- | --- | --- | --- | --- | --- | --- | --- | --- | --- | --- | --- | --- | --- | --- | --- | --- | --- |
| No. | (Author, year) | Title | abstract | keywords | Background | Objectives | Trial design | Participants | Interventions | Outcomes | Sample size | Randomization | Allocation | Implementation | Blinding | Statistical methods | Participant flow | Recruitment | Baseline data | Numbers analyzed | Outcomes | Ancillary analysis | Harms | Limitations | Generalizability | Interpretation | Registration | Protocol | Funding |
| 1 | (Gu et al, 2021) | △ | ○ | △ | ○ | ○ | △ | ○ | △ | △ | △ | × | × | × | × | △ | ○ | △ | ○ | ○ | △ | × | × | × | ○ | ○ | △ | × | ○ |
| 2 | (Li et al, 2020) | △ | ○ | △ | △ | ○ | △ | ○ | △ | △ | ○ | ○ | × | △ | △ | △ | ○ | △ | ○ | ○ | △ | × | × | △ | ○ | ○ | ○ | × | ○ |
| 3 | (Lin et al, 2020) | △ | ○ | △ | △ | △ | △ | ○ | △ | △ | △ | △ | × | × | △ | △ | ○ | △ | △ | ○ | △ | × | × | × | ○ | △ | × | × | × |
| 4 | (Hu et al, 2020) | △ | ○ | △ | △ | ○ | △ | △ | △ | △ | △ | ○ | × | × | × | △ | ○ | △ | △ | ○ | △ | × | ○ | × | △ | △ | × | × | × |
| 5 | (Wang et al, 2016) | △ | ○ | △ | △ | ○ | △ | ○ | △ | △ | △ | △ | × | × | × | △ | ○ | △ | △ | ○ | △ | × | × | × | ○ | ○ | × | × | ○ |
| 6 | (Zhao et al, 2011) | △ | ○ | △ | ○ | ○ | △ | ○ | △ | △ | △ | × | × | × | × | ○ | ○ | △ | ○ | ○ | △ | × | ○ | ○ | ○ | ○ | × | △ | × |
| 7 | (Cao, 2020) | △ | ○ | △ | △ | ○ | △ | ○ | △ | △ | △ | △ | × | × | × | △ | ○ | △ | △ | ○ | △ | × | × | × | ○ | ○ | × | × | × |
| 8 | (Ou et al, 2022) | △ | ○ | △ | △ | ○ | △ | ○ | △ | △ | △ | ○ | × | × | × | △ | ○ | △ | ○ | ○ | △ | × | ○ | × | ○ | ○ | △ | × | × |
| 9 | (Zhang et al, 2018) | △ | △ | △ | △ | ○ | △ | △ | △ | △ | △ | × | × | × | × | △ | ○ | × | △ | ○ | △ | × | ○ | × | ○ | × | × | × | × |
| 10 | (Zhu et al, 2016) | △ | ○ | △ | △ | ○ | △ | ○ | △ | △ | △ | × | × | × | × | △ | ○ | × | × | ○ | △ | × | × | × | △ | ○ | × | × | ○ |
| 11 | (Wang et al, 2015) | △ | ○ | △ | △ | ○ | △ | ○ | △ | △ | △ | ○ | × | × | × | △ | △ | △ | ○ | ○ | △ | × | △ | ○ | △ | ○ | × | × | ○ |
| 12 | (Chen, 2011) | △ | ○ | △ | △ | ○ | △ | △ | △ | △ | △ | × | × | × | × | △ | ○ | △ | △ | ○ | △ | × | × | × | ○ | ○ | × | × | × |
| 13 | (Yang et al, 2015) | △ | ○ | △ | △ | △ | △ | ○ | △ | △ | △ | × | × | × | × | △ | ○ | △ | △ | ○ | △ | × | ○ | × | ○ | △ | × | × | ○ |
| 14 | (Kong et al, 2016) | △ | ○ | △ | △ | ○ | △ | ○ | △ | △ | △ | × | × | × | × | △ | △ | △ | ○ | ○ | △ | × | △ | △ | ○ | ○ | × | × | ○ |
| 15 | (Su et al, 2022) | △ | × | △ | △ | △ | △ | △ | △ | △ | △ | × | × | × | × | △ | ○ | △ | △ | ○ | △ | × | × | × | ○ | △ | × | × | ○ |
| 16 | (Jiang, 2022) | △ | ○ | △ | ○ | ○ | △ | △ | △ | △ | △ | △ | × | × | × | △ | ○ | ○ | △ | ○ | △ | × | × | × | ○ | ○ | × | × | × |
| 17 | (Luo et al, 2021) | △ | × | △ | △ | △ | △ | ○ | △ | △ | △ | × | × | × | × | × | ○ | △ | △ | ○ | △ | × | × | × | ○ | △ | × | × | × |
| 18 | (Wang et al, 2021) | △ | ○ | △ | △ | ○ | △ | ○ | △ | △ | △ | × | × | × | × | △ | ○ | △ | △ | ○ | △ | × | △ | △ | ○ | △ | × | × | ○ |
| 19 | (He et al, 2020) | △ | ○ | △ | △ | ○ | △ | ○ | △ | △ | △ | × | × | × | × | △ | ○ | △ | ○ | ○ | △ | × | ○ | × | ○ | △ | × | × | ○ |
| 20 | (Luo et al, 2019) | △ | ○ | △ | △ | ○ | △ | △ | △ | △ | △ | × | × | × | × | △ | ○ | △ | △ | ○ | △ | × | ○ | × | ○ | ○ | × | × | × |
| 21 | (Liu, 2018) | △ | ○ | △ | △ | ○ | △ | △ | △ | △ | △ | × | × | × | × | △ | ○ | △ | × | △ | △ | × | × | × | ○ | △ | × | × | × |
| 22 | (Lin et al, 2018) | △ | ○ | △ | △ | △ | △ | △ | △ | △ | △ | × | × | × | × | △ | ○ | △ | △ | ○ | △ | × | × | × | ○ | ○ | × | × | × |
| 23 | (Li, 2016) | ○ | ○ | △ | △ | ○ | △ | △ | △ | △ | △ | ○ | × | × | × | △ | ○ | △ | ○ | × | △ | × | ○ | × | ○ | △ | × | × | ○ |
| 24 | (Song et al, 2016) | △ | ○ | △ | △ | ○ | △ | ○ | △ | △ | △ | ○ | × | × | × | △ | ○ | △ | ○ | ○ | △ | × | × | × | ○ | ○ | △ | × | × |
| 25 | (Liu et al, 2016) | △ | ○ | △ | △ | ○ | △ | ○ | △ | △ | △ | × | × | × | × | △ | ○ | △ | ○ | × | △ | × | ○ | △ | ○ | △ | × | × | × |
| 26 | (Li, 2016) | △ | ○ | △ | △ | ○ | △ | ○ | △ | △ | △ | ○ | × | × | × | ○ | ○ | △ | △ | ○ | △ | ○ | ○ | × | ○ | ○ | × | × | ○ |
| 27 | (Zhang et al, 2016) | △ | ○ | △ | △ | △ | △ | ○ | △ | △ | △ | × | × | × | × | △ | ○ | △ | △ | ○ | △ | × | × | × | ○ | △ | × | × | × |
| 28 | (Liang et al, 2016) | △ | ○ | △ | △ | △ | △ | ○ | △ | △ | △ | × | × | × | × | △ | ○ | × | △ | ○ | △ | × | ○ | × | ○ | ○ | × | × | × |
| 29 | (Sun, 2015) | △ | ○ | △ | △ | △ | △ | △ | △ | △ | △ | × | × | × | × | △ | ○ | △ | △ | ○ | △ | × | × | × | ○ | △ | × | × | × |
| 30 | (Li et al, 2011) | △ | ○ | △ | △ | ○ | △ | ○ | △ | △ | △ | × | × | × | × | △ | ○ | △ | △ | ○ | △ | × | ○ | × | ○ | ○ | △ | × | × |
| 31 | (Huang, 2001) | △ | × | △ | △ | ○ | △ | △ | △ | △ | △ | × | × | × | × | △ | ○ | △ | △ | ○ | △ | × | × | × | ○ | △ | × | × | × |
| 32 | (Cao et al, 2022) | △ | ○ | △ | △ | ○ | △ | ○ | △ | △ | △ | △ | × | × | × | △ | ○ | △ | △ | ○ | △ | × | × | × | ○ | ○ | × | × | ○ |
| 33 | (Zhang et al, 2019) | △ | △ | △ | △ | ○ | △ | ○ | △ | △ | △ | ○ | × | × | × | △ | ○ | △ | △ | △ | △ | × | × | × | ○ | △ | × | × | × |
| 34 | (Wang, 2019) | △ | ○ | △ | △ | △ | △ | △ | △ | △ | △ | △ | × | × | × | △ | ○ | △ | △ | ○ | △ | × | × | × | ○ | △ | × | × | × |
| 35 | (Yao, 2019) | △ | ○ | △ | △ | ○ | △ | ○ | △ | △ | △ | × | × | × | × | △ | ○ | △ | △ | ○ | △ | × | × | × | ○ | △ | × | × | × |
| 36 | (Chen et al, 2019) | △ | ○ | △ | △ | ○ | △ | ○ | △ | △ | △ | × | × | × | × | △ | ○ | △ | △ | ○ | △ | × | × | × | ○ | △ | × | × | ○ |
| 37 | (Wang, 2018) | △ | × | △ | △ | ○ | △ | △ | △ | △ | △ | × | × | × | × | △ | ○ | △ | △ | ○ | △ | × | × | △ | △ | △ | × | × | × |
| 38 | (Guo et al, 2017) | △ | ○ | △ | △ | ○ | △ | ○ | △ | △ | △ | ○ | × | × | × | △ | ○ | △ | △ | ○ | △ | × | × | △ | ○ | ○ | × | × | ○ |
| 39 | (Gu et al, 2009) | △ | × | × | △ | △ | △ | △ | △ | △ | △ | △ | × | × | × | △ | ○ | △ | △ | ○ | △ | × | × | × | △ | △ | × | × | × |
| 40 | (Liu et al, 2014) | △ | ○ | △ | △ | △ | △ | ○ | △ | △ | △ | × | × | × | × | △ | ○ | △ | △ | ○ | △ | × | × | × | ○ | △ | × | × | × |
| 41 | (Li et al, 2013) | △ | ○ | △ | △ | ○ | △ | ○ | △ | △ | △ | × | × | × | × | △ | ○ | △ | ○ | ○ | △ | × | ○ | × | ○ | △ | × | × | ○ |
| 42 | (Wang et al, 2016) | △ | ○ | △ | △ | △ | △ | ○ | △ | △ | △ | △ | × | × | × | △ | ○ | △ | △ | ○ | △ | × | × | × | ○ | ○ | × | × | ○ |
| 43 | (Cai, 2016) | △ | ○ | △ | △ | ○ | △ | △ | △ | △ | △ | × | × | × | × | △ | ○ | △ | △ | ○ | △ | × | × | × | ○ | △ | × | × | × |
| 44 | (Ning et al, 2020) | △ | ○ | △ | △ | ○ | △ | ○ | △ | △ | △ | ○ | × | × | × | △ | ○ | △ | ○ | ○ | △ | × | ○ | × | ○ | ○ | × | × | ○ |
| 45 | (Yang, 2020) | △ | ○ | △ | △ | ○ | △ | ○ | △ | △ | △ | ○ | × | × | × | △ | ○ | △ | △ | ○ | △ | × | × | × | ○ | △ | △ | × | × |
| 46 | (Shan et al, 2020) | △ | ○ | △ | △ | ○ | △ | ○ | △ | △ | △ | × | × | × | × | △ | ○ | △ | △ | ○ | △ | × | × | × | ○ | ○ | × | × | × |
| 47 | (Wu, 2018) | △ | ○ | △ | △ | ○ | △ | △ | △ | △ | △ | × | × | × | × | △ | ○ | △ | △ | ○ | △ | × | × | × | ○ | △ | × | × | × |
| 48 | (Shi, 2017) | △ | ○ | △ | △ | ○ | △ | ○ | △ | △ | △ | ○ | × | × | × | △ | ○ | △ | △ | × | △ | × | × | × | ○ | △ | △ | × | × |
| 49 | (Zhang et al, 2017) | △ | ○ | △ | △ | ○ | △ | ○ | △ | △ | △ | ○ | × | × | × | △ | ○ | △ | △ | △ | △ | × | ○ | × | ○ | △ | × | × | × |
| 50 | (Wang et al, 2015) | △ | ○ | △ | △ | △ | △ | △ | △ | △ | △ | × | × | × | × | △ | ○ | △ | △ | ○ | △ | × | × | × | ○ | ○ | × | × | × |
| 51 | (Lu, 2014) | △ | △ | △ | △ | △ | △ | △ | △ | △ | △ | × | × | × | × | × | ○ | △ | △ | ○ | △ | × | × | × | △ | △ | × | × | × |
| 52 | (Zhang et al, 2019) | △ | ○ | △ | △ | ○ | △ | △ | △ | △ | △ | ○ | × | × | × | △ | ○ | △ | △ | ○ | △ | × | × | × | △ | △ | × | × | × |
| 53 | (Zhang et al, 2017) | △ | ○ | △ | △ | ○ | △ | ○ | △ | △ | △ | ○ | × | × | × | △ | ○ | △ | ○ | ○ | △ | × | ○ | × | ○ | △ | × | × | × |
| 54 | (Wu et al, 2014) | △ | ○ | △ | △ | ○ | △ | △ | △ | △ | △ | ○ | × | × | × | △ | ○ | △ | △ | × | △ | × | × | × | △ | ○ | △ | × | × |
| 55 | (Feng, 2014) | △ | ○ | △ | △ | ○ | △ | △ | △ | △ | △ | × | × | × | × | △ | ○ | △ | △ | ○ | △ | × | ○ | × | ○ | ○ | △ | × | × |
| 56 | (Zhang et al, 2012) | △ | ○ | △ | △ | △ | △ | △ | △ | △ | △ | × | × | × | × | △ | ○ | △ | △ | △ | △ | × | ○ | × | ○ | △ | × | × | × |
| 57 | (Li, 2015) | △ | ○ | △ | △ | ○ | △ | ○ | △ | △ | △ | ○ | × | × | × | △ | ○ | △ | △ | ○ | △ | × | × | ○ | ○ | ○ | × | × | ○ |
| 58 | (Yang et al, 2018) | △ | ○ | △ | △ | ○ | △ | △ | △ | △ | △ | × | × | × | × | △ | ○ | △ | △ | × | △ | × | × | × | △ | △ | × | × | × |
| 59 | (Ou et al, 2016) | △ | ○ | △ | △ | ○ | △ | △ | △ | △ | △ | × | × | × | × | △ | ○ | △ | △ | ○ | △ | × | ○ | × | ○ | ○ | × | × | × |
| 60 | (Zhao, 2015) | △ | ○ | △ | △ | △ | △ | ○ | △ | △ | △ | × | × | × | × | △ | ○ | △ | △ | ○ | △ | × | × | × | △ | △ | × | × | × |
| 61 | (Leng, 2015) | △ | ○ | △ | △ | ○ | △ | △ | △ | △ | △ | × | × | × | × | △ | ○ | △ | △ | ○ | △ | × | × | × | △ | △ | × | × | × |
| 62 | (Jing et al, 2010) | △ | × | △ | △ | △ | △ | △ | △ | △ | △ | △ | × | × | × | △ | ○ | △ | △ | ○ | △ | × | × | × | △ | △ | × | × | × |
| 63 | (Huang, 2012) | △ | ○ | △ | △ | △ | △ | △ | △ | △ | △ | × | × | × | × | △ | ○ | △ | △ | ○ | △ | × | ○ | × | △ | △ | × | × | × |
| 64 | (Wei, 2016) | △ | ○ | △ | △ | △ | △ | ○ | △ | △ | △ | ○ | × | × | × | △ | ○ | △ | △ | ○ | △ | × | ○ | × | ○ | ○ | × | × | × |
| 65 | (Wu et al, 2014) | △ | ○ | △ | △ | △ | △ | △ | △ | △ | △ | × | × | × | × | △ | ○ | △ | △ | ○ | △ | × | × | × | △ | △ | × | × | × |
| 66 | (Dai et al, 2013) | △ | ○ | △ | △ | △ | △ | △ | △ | △ | △ | × | × | × | × | △ | ○ | △ | △ | ○ | △ | × | ○ | × | ○ | ○ | × | × | × |
| 67 | (Huang et al, 2013) | △ | ○ | △ | △ | △ | △ | ○ | △ | △ | △ | ○ | × | × | × | △ | ○ | △ | △ | △ | △ | × | ○ | × | ○ | △ | × | × | × |
| 68 | (Wu, 2014) | △ | ○ | △ | △ | ○ | △ | △ | △ | △ | △ | ○ | × | × | × | △ | ○ | △ | △ | △ | △ | × | × | × | ○ | △ | × | × | × |
| 69 | (Liang et al, 2012) | △ | ○ | △ | △ | ○ | △ | △ | △ | △ | △ | × | × | × | × | △ | ○ | △ | △ | ○ | △ | × | × | × | △ | ○ | × | × | ○ |
| 70 | (Wei, 2021) | △ | ○ | △ | △ | ○ | △ | △ | △ | △ | △ | × | × | × | × | △ | ○ | △ | △ | ○ | △ | × | × | × | △ | △ | × | × | × |
| 71 | (Cui et al, 2022) | ○ | ○ | △ | △ | ○ | △ | ○ | △ | △ | △ | ○ | × | × | × | △ | ○ | △ | △ | ○ | △ | × | ○ | × | ○ | ○ | ○ | × | ○ |
| 72 | (Guglielmo et al, 2020) | ○ | ○ | △ | ○ | ○ | ○ | ○ | △ | ○ | ○ | × | × | × | △ | ○ | ○ | △ | ○ | △ | ○ | ○ | × | ○ | ○ | ○ | △ | × | × |
| 73 | (Jeong et al, 2010) | ○ | ○ | △ | △ | ○ | ○ | ○ | ○ | ○ | ○ | ○ | × | × | × | △ | ○ | △ | ○ | ○ | ○ | × | ○ | ○ | ○ | ○ | △ | △ | ○ |
| 74 | (Gu et al, 2010) | △ | ○ | △ | △ | △ | △ | ○ | △ | △ | △ | × | × | × | × | ○ | ○ | △ | △ | △ | △ | ○ | × | × | ○ | ○ | × | × | × |
| 75 | (Oliveira et al, 2011) | △ | ○ | × | ○ | △ | ○ | ○ | △ | ○ | △ | ○ | ○ | ○ | △ | ○ | ○ | △ | ○ | ○ | ○ | ○ | ○ | ○ | ○ | ○ | △ | × | ○ |
| 76 | (Kim et al, 2020) | ○ | ○ | △ | ○ | ○ | ○ | ○ | ○ | ○ | ○ | ○ | ○ | △ | △ | ○ | ○ | △ | ○ | ○ | ○ | ○ | ○ | △ | ○ | ○ | ○ | ○ | ○ |
| 77 | (Barton et al, 2010) | ○ | ○ | △ | ○ | ○ | △ | △ | ○ | ○ | △ | ○ | ○ | × | △ | △ | ○ | △ | ○ | △ | ○ | × | ○ | △ | ○ | ○ | × | × | ○ |
| 78 | (Sette et al, 2018) a | ○ | △ | ○ | △ | △ | △ | △ | △ | ○ | ○ | × | × | × | × | △ | ○ | △ | ○ | △ | ○ | × | ○ | × | ○ | ○ | ○ | ○ | × |
|  | (Sette et al, 2018) b | ○ | △ | ○ | △ | △ | △ | △ | △ | ○ | ○ | × | × | × | × | △ | ○ | △ | ○ | △ | ○ | × | ○ | × | ○ | ○ | ○ | ○ | × |
| 79 | (Lee et al, 2021) | ○ | ○ | △ | △ | ○ | ○ | ○ | ○ | ○ | ○ | ○ | ○ | ○ | △ | ○ | ○ | △ | ○ | ○ | ○ | × | ○ | ○ | ○ | ○ | ○ | ○ | ○ |
| 80 | (Barton et al, 2013) | ○ | ○ | × | ○ | ○ | △ | ○ | ○ | ○ | ○ | ○ | × | × | × | △ | ○ | △ | ○ | ○ | ○ | × | ○ | × | ○ | ○ | ○ | × | ○ |
| 81 | (Costa et al, 2009) | ○ | ○ | × | △ | ○ | △ | △ | △ | ○ | △ | × | × | × | × | ○ | ○ | △ | △ | ○ | ○ | ○ | ○ | △ | ○ | ○ | ○ | × | ○ |

Notes: ○: fully met the CONSORT-CHM criteria; △: partially met the CONSORT-CHM criteria; ×: not met the CONSORT-CHM criteria.


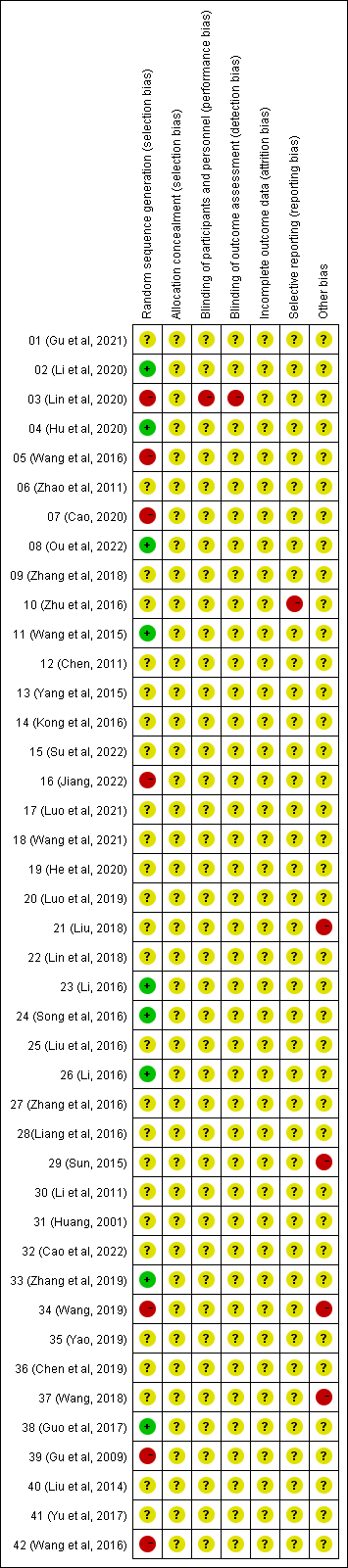


Additional document 2. Figure S1a. Risk of bias summary: review authors' judgements about each risk of bias item for each included study (Study No. 01-42)


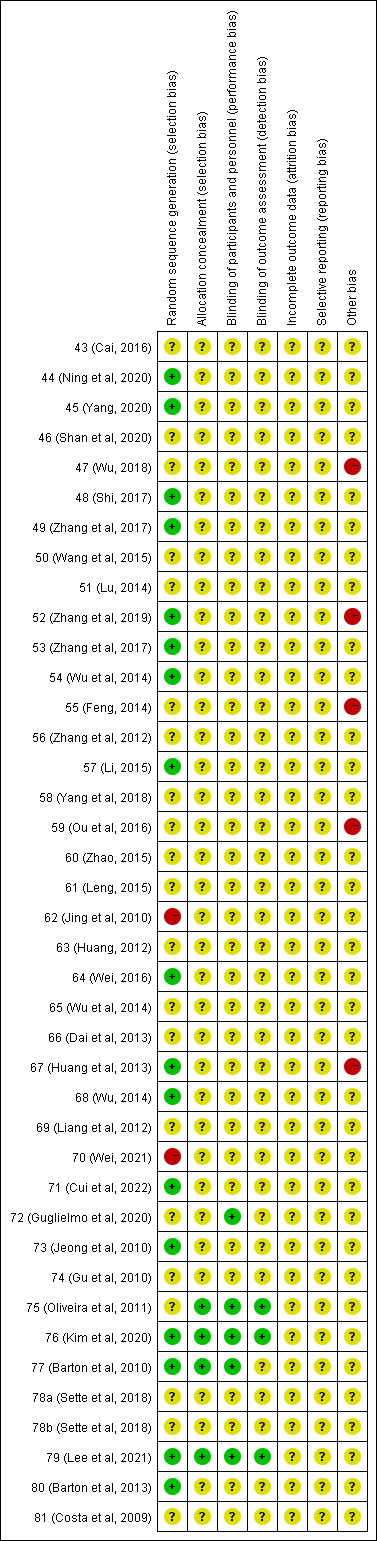


Additional document 2. Figure S1b. Risk of bias summary: review authors' judgements about each risk of bias item for each included study (Study No. 43-81)
